# Mapping of Variable DNA Methylation Across Multiple Cell Types Defines a Dynamic Regulatory Landscape of the Human Genome

**DOI:** 10.1534/g3.115.025437

**Published:** 2016-02-16

**Authors:** Junchen Gu, Michael Stevens, Xiaoyun Xing, Daofeng Li, Bo Zhang, Jacqueline E. Payton, Eugene M. Oltz, James N. Jarvis, Kaiyu Jiang, Theodore Cicero, Joseph F. Costello, Ting Wang

**Affiliations:** * Department of Genetics, Center for Genome Sciences and Systems Biology, Washington University School of Medicine, St. Louis, Missouri 63108; † Department of Computer Science and Engineering, Washington University in St. Louis, Missouri 63130; ‡ Department of Developmental Biology, Washington University School of Medicine, St. Louis, Missouri 63108; § Department of Pathology and Immunology, Washington University School of Medicine, St. Louis, Missouri 63110; ** Department of Pediatrics, University of Buffalo, New York 14203; †† Department of Psychiatry, Washington University School of Medicine, St. Louis, Missouri 63110; ‡‡ Brain Tumor Research Center, Department of Neurosurgery, Helen Diller Family Comprehensive Cancer Center, University of California, San Francisco, California 94143

**Keywords:** DNA methylomes, methylCRF, methylation dynamics, regulatory elements

## Abstract

DNA methylation is an important epigenetic modification involved in many biological processes and diseases. Many studies have mapped DNA methylation changes associated with embryogenesis, cell differentiation, and cancer at a genome-wide scale. Our understanding of genome-wide DNA methylation changes in a developmental or disease-related context has been steadily growing. However, the investigation of which CpGs are variably methylated in different normal cell or tissue types is still limited. Here, we present an in-depth analysis of 54 single-CpG-resolution DNA methylomes of normal human cell types by integrating high-throughput sequencing-based methylation data. We found that the ratio of methylated to unmethylated CpGs is relatively constant regardless of cell type. However, which CpGs made up the unmethylated complement was cell-type specific. We categorized the 26,000,000 human autosomal CpGs based on their methylation levels across multiple cell types to identify variably methylated CpGs and found that 22.6% exhibited variable DNA methylation. These variably methylated CpGs formed 660,000 variably methylated regions (VMRs), encompassing 11% of the genome. By integrating a multitude of genomic data, we found that VMRs enrich for histone modifications indicative of enhancers, suggesting their role as regulatory elements marking cell type specificity. VMRs enriched for transcription factor binding sites in a tissue-dependent manner. Importantly, they enriched for GWAS variants, suggesting that VMRs could potentially be implicated in disease and complex traits. Taken together, our results highlight the link between CpG methylation variation, genetic variation, and disease risk for many human cell types.

DNA methylation refers to the addition of a methyl group at the C5 position of cytosine in DNA sequences. Methylation on cytosine can occur in different sequence contexts but largely in a CpG dinucleotide context (Fazzari and Greally 2004). Proper establishment of DNA methylation early in embryogenesis is vital for normal development in many organisms (Law and Jacobsen 2010). DNA methylation plays a pivotal role in genomic imprinting and X-chromosome inactivation, where methylation of one parental allele suppresses its expression and leads to monoallelic gene expression (Reik and Lewis 2005). In addition, epigenetic modifications of the chromatin, including DNA methylation and histone modifications, orchestrate heritable, cell type- and developmental stage-specific gene expression in vertebrates (Robertson 2005; Portela and Esteller 2010).

Since the advent and wide adaptation of next-generation sequencing in epigenomic studies (Harris *et al.* 2010), many studies have provided insights into the functions of DNA methylation at a genome-wide scale. We now have a catalog of DNA methylomes of many cell types in different organisms (Lister *et al.* 2009, 2013; Shen *et al.* 2012; Kobayashi *et al.* 2013; Zhang *et al.* 2013; Lee *et al.* 2015). While the majority of DNA methylation remains stable once a cell is fully differentiated, DNA methylation undergoes changes during embryogenesis, cell differentiation, tissue development, aging, and disease progression (Jiang *et al.* 2013; Meissner *et al.* 2008; Laurent *et al.* 2010). Recently, the Roadmap Epigenomics Consortium generated and analyzed a large number of reference epigenomic profiles including histone modifications, DNA accessibility, DNA methylation, and gene expression across a large number of cell and tissue types. Collectively, the integrated analysis predicted many elements distal to genes that colocalized with various histone modifications, DNA methylation patterns, and relevant transcription factor binding at either open or closed chromatin regions, and many of them could have gene regulatory functions (Kundaje *et al.* 2015). In particular, loss of DNA methylation accompanied a gain of histone modifications indicative of a poised chromatin state and a gain of lineage-relevant transcription factor binding during differentiation of human embryonic stem (ES) cells into different lineages (Tsankov *et al.* 2015). Furthermore, in agreement with a similar finding in mice, transcription factor binding correlated with a decrease in local DNA methylation (Stadler *et al.* 2011). In a study on neural differentiation from ES cells, different patterns of DNA methylation changes were observed during consecutive stages of neural progenitors derived from ES cells (Ziller *et al.* 2015). Additionally, allelic gene expression was correlated with promoter DNA methylation in differentiated lineages from H1 ES cells and certain allelic enhancers showed differential DNA methylation (Dixon *et al.* 2015). By analyzing primary breast cell types, DNA methylation was found to be a stable signature of expressed exons in luminal and myoepithelial cells (Gascard *et al.* 2015). During development, it was found that cell origin has a stronger impact than tissue environment in determining genome-wide DNA methylation patterns (Lowdon *et al.* 2014). Lastly, intermediate methylation represented a conserved signature of gene regulation and exon usage through an integrative analysis of many cell types (Elliott *et al.* 2015).

While much effort has been focused on identifying DNA methylation changes associated with specific treatment, environmental stimuli, or with differentiation, we know relatively little about the systematic patterns of DNA methylation across many cell or tissue types under physiologically normal conditions. One study analyzed 42 human Whole Genome Bisulfite Sequencing (WGBS) libraries and identified 21.8% of dynamically regulated autosomal CpGs (Ziller *et al.* 2013). Another recent study found that 15.4% of CpGs are strongly differentially methylated among 36 human DNA methylomes (Schultz *et al.* 2015). While these studies provided valuable resources and insights, the number of single-CpG-resolution DNA methylomes and the number of cell types profiled are limited. We still do not have a complete picture of which fraction of the methylome is stable and the extent to which variable DNA methylation contributes to genome regulation.

In this study, we set out to define the variably methylated regions of the human DNA methylome. We combined a large collection of genome-wide DNA methylation profiles spanning multiple cell and tissue types using two complementary methods, Methylation Dependent ImmunoPrecipitation followed by sequencing (MeDIP-seq) and Methylation-sensitive Restriction Enzyme digestion followed by sequencing (MRE-seq) (Li *et al.* 2014). Using a recently developed conditional random fields-based algorithm, methylCRF (Stevens *et al.* 2013), we estimated 54 single-CpG-resolution DNA methylomes. Consistent with previous reports, we demonstrated that our DNA methylomes are equal in quality to those profiled by Whole Genome Bisulfite Sequencing (WGBS) but much more cost-effective (Stevens *et al.* 2013; Lee *et al.* 2015). In-depth analysis of these high-resolution DNA methylomes revealed that for each cell type, around 11% of autosomal CpGs are unmethylated, of which 7% are constantly unmethylated; overall 22.6% show variable methylation across the cell types we investigated. Combining the variably methylated CpGs into regions, we characterized the features of these regions and their colocalization with various regulatory elements such as enhancer-associated histone modifications, transcription factor binding sites, and disease-associated GWAS (genome-wide association study) variants, and uncovered many important functions that these dynamic regions might possess.

## Materials and Methods

### Sample collection

All samples were collected from normal individuals and prepared in a timely fashion to ensure the isolation of high-quality genomic DNA. Tonsil, endometrium, granulocytes, and monocytes were processed as previously described (Jarvis *et al.* 2009; Zhang *et al.* 2014; Koues *et al.* 2015). Whole blood was pooled venous blood samples from normal male individuals. All other samples were processed in the Roadmap Epigenomics Project and the sample collections have been previously described (Lowdon *et al.* 2014; Zhang *et al.* 2013).

### Data processing and methylCRF prediction

MeDIP-seq and MRE-seq data were aligned using BWA (Li and Durbin 2009) against human genome reference hg19 and then processed with methylCRF to generate single CpG methylation predictions for each of the 54 samples (Stevens *et al.* 2013). For the rest of the analysis, only autosomal CpGs were considered.

### CpG categorization

Autosomal CpGs were initially grouped into four biologically motivated categories based on their distribution of DNA methylation values across the 54 samples: constitutively unmethylated (U) if all values were below 30%, intermediately methylated (I) if at least 50 libraries had values between 30–70%, constitutively methylated (M) if all values were above 70%, and variably methylated if there were was a gap of at least 40% between the third highest and the third lowest methylation values. The remaining CpGs were then assigned one of the categories using k-Nearest Neighbors (k-NN) with a k of 4. k-NN was iterated 10 times, once for each category, to allow convergence.

For enrichment analysis, we merged CpGs within 500 bp of each other with the same category label to create regions. However, CpGs crossing blacklisted regions were not merged (The ENCODE Project Consortium 2012).

For visual inspection on a genome browser, we created two tracks by segmenting the genome based on CpG methylation patterns across all 54 samples. For the CpG Regions track, we merged CpGs within 500 bp of each other for each CpG category, in the same way as for enrichment analysis. For the CpG Regions Extended track, we extended the CpG Regions in both directions so that the neighboring regions were next to each other; for regions between CpGs greater than 1000 bp apart, we created a CpG "desert” state “D”. The “D” state started 50 bp away from each CpG. Finally, we designated blacklisted regions with “B.”

### Genomic distribution of VMRs

Transcription start sites (TSSs) and information about other genomic features were downloaded from the UCSC genome browser (hg19) (Kent *et al.* 2002). The promoter was defined as 2000 bp regions upstream of the TSS for each gene. Intergenic regions were defined as intervals between the end of gene bodies and the beginning of promoters. For genomic distribution analysis, background regions of matching number and size with VMRs were randomly selected from the human genome.

### Determining cell type-specific hypomethylated VMRs

To call hypomethylated VMRs for each cell type, we first calculated the average DNA methylation levels of all the CpGs located in a VMR for each sample and then averaged the DNA methylation levels for samples of the same cell type. We then called a VMR hypomethylated in a cell type if the average cell type DNA methylation level was below 30%. To ensure cell type specificity, we further required that a VMR could only be called cell type-specific hypomethylated in no more than three cell types.

### TFBS ChIP-seq peak fold enrichment

Transcription factor binding site (TFBS) ChIP-seq narrow peaks data were downloaded from ENCODE (The ENCODE Project Consortium 2012) and used in the transcription factor peak fold enrichment analysis. The fold enrichment of TFBS ChIP-seq peaks in VMRs were calculated using the number of VMRs overlapping ChIP-seq peaks divided by the number of size-matched randomly selected regions overlapping the same TFBS ChIP-seq peaks. We used BEDTools utilities to calculate the intersection (Quinlan and Hall 2010). We then applied ANOVA with FDR correction to identify tissue type-specific TFBS peak fold enrichment for each transcription factor.

### Transcription factor binding motif enrichment analysis

HOMER was used to search for known transcription factor binding motifs in hypomethylated VMRs according to the instructions provided on the website with default parameters (Heinz *et al.* 2010). The top five motifs and corresponding enrichment score p-values were examined for functional relevance. For tissues with multiple cell types, the median value of all the p-values from different cells was used to plot the enrichment (Supplemental Material, Figure S5).

### ChromHMM state enrichment calculation and histone modification ChIP-seq peak calling

Core15-state ChromHMM annotation files were obtained from the Roadmap Epigenomics Consortium (Kundaje *et al.* 2015). The fold enrichment of ChromHMM states in VMRs were calculated using the number of VMRs whose centers overlap with a state divided by the number of size-matched randomly selected regions from the genome overlapping the same chromatin state. The resulting fold enrichment values were log2 transformed and visualized with a heat map. Histone modification ChIP-seq data were downloaded from GEO (GEO ID: GSE16368). Processed BED files were used for each ChIP-seq dataset. Histone peaks were called using SICER with the default parameters against hg19 (Zang *et al.* 2009).

### Enhancer analysis and functional enrichment of genes

VISTA enhancer databases were downloaded from the VISTA Enhancer Browser (Visel *et al.* 2007). Human positively validated enhancer regions were used in the analysis and built into a custom browser track. Gene Ontology enrichment analysis of genes near VMRs was performed using the GREAT analysis tool (McLean *et al.* 2010) with the default settings for defining gene regulatory domains to associate genes to VMRs.

### GWAS variants

GWAS variants data were downloaded from the GWAS Catalog of the National Human Genome Research Institute (Hindorff *et al.* 2014). The fold enrichment of GWAS variants in hypomethylated VMRs were calculated using the number of GWAS variants overlapping hypomethylated VMRs divided by the number of GWAS variants overlapping size-matched randomly selected regions. For the relevant GWAS traits enrichment, GWAS traits linked to the identified variants were used to calculate fold enrichment in VMRs against all the occurrences in the GWAS catalog by hypergeometric test. Multiple testing corrections on the hypergeometric p-values were done by the FDR method for each cell type.

### Concordance calculation between methylCRF and WGBS

Concordances of DNA methylation levels between methylCRF and WGBS were calculated by counting the number of CpGs with methylation level differences between methylCRF and WGBS less than 25%. Then, the percentage of CpGs meeting the above criterion was calculated as the concordance. For WGBS, a minimum read coverage of 10 was used to select CpGs.

### Saturation analysis

We randomly selected n number of samples (n goes from 6–53) and identified variably methylated CpGs with 10 iterations, and then the percentage of variably methylated CpGs were plotted against the number of samples selected.

### Data availability and visualization

All the methylCRF prediction custom tracks are displayed using the WashU Epigenome Browser (Zhou *et al.* 2011). The read alignment and processed data for MeDIP-seq and MRE-seq from this publication have been submitted to the GEO database and assigned the identifier GSE69894. Relevant variably methylated CpGs and regions data and browser visualization can be accessed on the website: http://epigenome.wustl.edu/methylomes.

## Results

### Global analysis of 54 methylomes revealed a consistent proportion of unmethylated CpGs across cell types coupled with distinct cell-type specific signatures

In total, we interrogated the DNA methylomes of 54 normal human primary cell samples (21 cell/tissue types) including, fetal brain, cortex-derived and ganglionic eminence-derived neurosphere cells, fibroblasts, keratinocytes, skin melanocytes, luminal epithelial cells, myoepithelial and stem breast cells, CD4 memory cells, CD4 naïve cells, CD14 monocytes, whole blood, granulocytes, peripheral blood mononuclear cells (PBMC), and endometrium. For a complete list of cell and tissue types, refer to Table S1.

**Figure 1:**
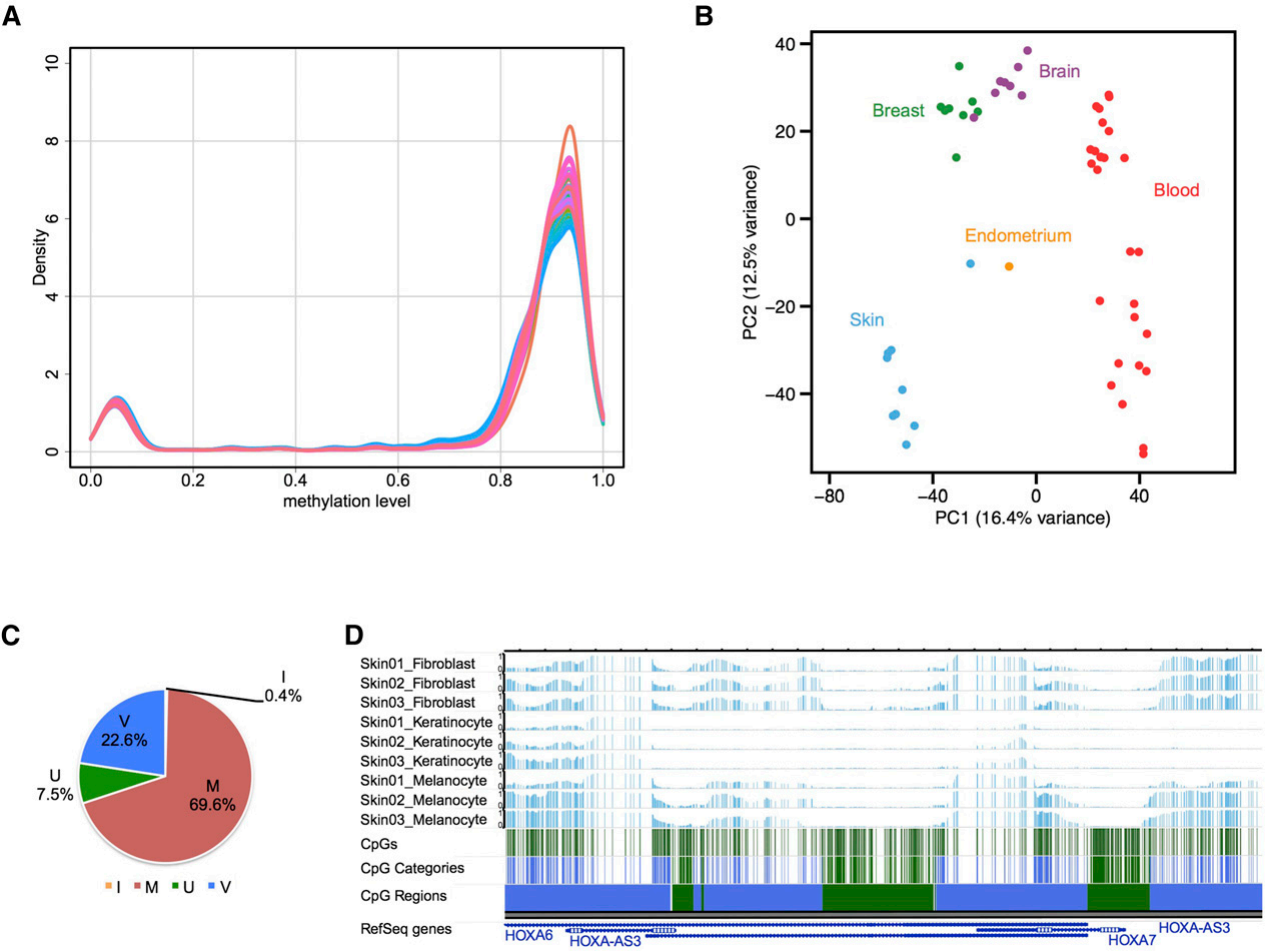
Characterization of autosomal CpG methylation patterns across cell types and identification of variably methylated CpGs. (A) Density plot of genome-wide DNA methylation levels of all CpGs for each of the 54 methylCRF (conditional random fields-based algorithm) predictions. (B) Principal Component Analysis of average CpG methylation levels for 1 kb genomic windows across 54 methylCRF predictions. Note the clustering of methylomes by tissue type. (C) Percentage of autosomal CpGs in each category after applying a k-NN (k-Nearest Neighbors) algorithm. M: constitutively methylated CpGs; I: constitutively intermediately methylated CpGs; U: constitutively unmethylated CpGs; V: variably methylated CpGs. (D) An example WashU Epigenome Browser view of a genomic region showing different categories of CpGs and the corresponding combined regions. Note the depletion of methylation around the HOXA7 gene specifically in skin keratinocytes. Three tracks each for skin fibroblasts, keratinocytes, and melanocytes; CpG track, CpG categories, and the resulting regions are displayed. In the CpG Categories and CpG Regions tracks, green indicates constitutively unmethylated CpGs and regions and blue indicates variably methylated CpGs and regions.

methylCRF estimates complete DNA methylomes at single-CpG-resolution by combining MeDIP-seq and MRE-seq. For all the following analyses, only autosomal CpGs were considered. We looked at several metrics of the overall state of methylation in all cell types. The average DNA methylation levels of all the autosomal CpGs for each cell type ranged from 77.5–81.7% and displayed small levels of variation among tissue types and similar levels of methylation among different cell types of the same tissue type profiled in this study (Table S1 and Figure S1A). For all cell types, the overall distribution of the DNA methylation levels followed an expected pattern where the majority of CpGs were either highly methylated (82.4–87.8% of the total CpGs) or unmethylated (9.7–12.5% of the total CpGs), and a small percentage (2.4–5.6%) of CpGs were intermediately methylated (Figure 1A, Figure S1B, and Table S1).

Multiple studies support the hypothesis that low DNA methylation can act as a signature of gene regulation (Stadler *et al.* 2011; Zhang *et al.* 2013; Tsankov *et al.* 2015). We thus thought to identify the potential of each CpG to have a regulatory role in gene regulation by calculating the lowest possible DNA methylation level for each CpG. We found that 6,769,260 autosomal CpGs (25.4% of total) have their lowest DNA methylation level below 30% (Figure S1C). This finding suggests that, although the distribution of DNA methylation maintains the same pattern where roughly 11% of CpGs are unmethylated (average of the percentage of unmethylated CpGs among all cell types) for any given cell type, 25.4% of the CpGs could be unmethylated and potentially have regulatory functions.

Different cell types have distinct methylation signatures. We confirmed that our data supports this notion. By applying the Principle Component Analysis (PCA) and hierarchical clustering analysis on our DNA methylomes at 1 kb resolution, we found that the separation and clustering of samples were defined by tissue types as well as by cell types (Figure 1B and Figure S1D). In particular, different breast cell type methylomes had notable similarity to each other whereas blood cell types were not as closely clustered, reflecting tissue type-dependent variability among samples in their global DNA methylation patterns. This variability could be due to epigenomic variability within the same cell type, heterogeneity in cell preparation, or their genetic background.

The average methylation levels for different genomic features in all the cell types examined shared the expected pattern of relatively low average methylation levels in promoter regions compared to gene bodies (exons and introns) or intergenic regions. Low methylation levels were particularly evident in high CpG promoters (Figure S1, E and F).

### Classification of CpGs based on their methylation patterns across all samples identified 22.6% of CpGs being variably methylated

Our data revealed that the overall fractions of methylated or unmethylated CpGs across the 54 methylomes were remarkably similar. However, for any given CpG, its methylation level can be either stable or variable across the samples. To analyze this characteristic, we categorized CpGs into biologically informative types. We used simple and conservative notions of constitutively methylated (M), unmethylated (U), and intermediately methylated (I), as well as variably methylated (V), CpGs to classify CpGs using 70% and 30% as cutoffs (See *Materials and Methods* for details). We were able to assign 21.7 million CpGs based on these criteria. We then used the categorized CpGs as seed sets for a k-NN algorithm to classify the remaining CpGs as well as to refine the category boundaries. This categorization resulted in 22.6% of CpGs being variably methylated along with 70% of CpGs being constitutively methylated. The fact that 70% of the autosomal CpGs are stably methylated (≥ 70% methylation) in all the 54 samples profiled in this study is in line with the notion that DNA methylation is a stable epigenetic mark across different human cell types. Of constitutively methylated CpGs, 61% were located in repeats and 82% of CpGs in repeats were constitutively methylated (Figure S2, A and B). On the other hand, the majority (75%) of constitutively unmethylated CpGs were located in CpG islands, and 74% of all the CpGs in CpG islands were constitutively unmethylated CpGs (Figure S2, C and D). These results agree with known patterns of DNA methylation with respect to repeats and CpG islands. After we categorized each CpG, we segmented the genome into regions of different DNA methylation patterns by combining CpGs of the same category that are within 500 bp of each other into corresponding regions, which can be viewed on the Washington University Epigenome Browser (*Materials and Methods*, Figure 1D, and Figure S3) (Zhou *et al.* 2011).

### A total of 85% of VMRs were less than 1 kb in length

Many studies have identified differentially methylated regions and linked the differential methylation to regulation of gene expression and cell type differentiation (Zhang *et al.* 2013; Lowdon *et al.* 2014). We thus hypothesized that variably methylated CpGs play a role in regulating gene expression in different cell types and focused our analysis on CpGs exhibiting variable DNA methylation levels across cell types. Overall, we defined 6,014,012 CpGs as variably methylated CpGs, and 663,916 regions as VMRs (Table S2).

**Figure 2:**
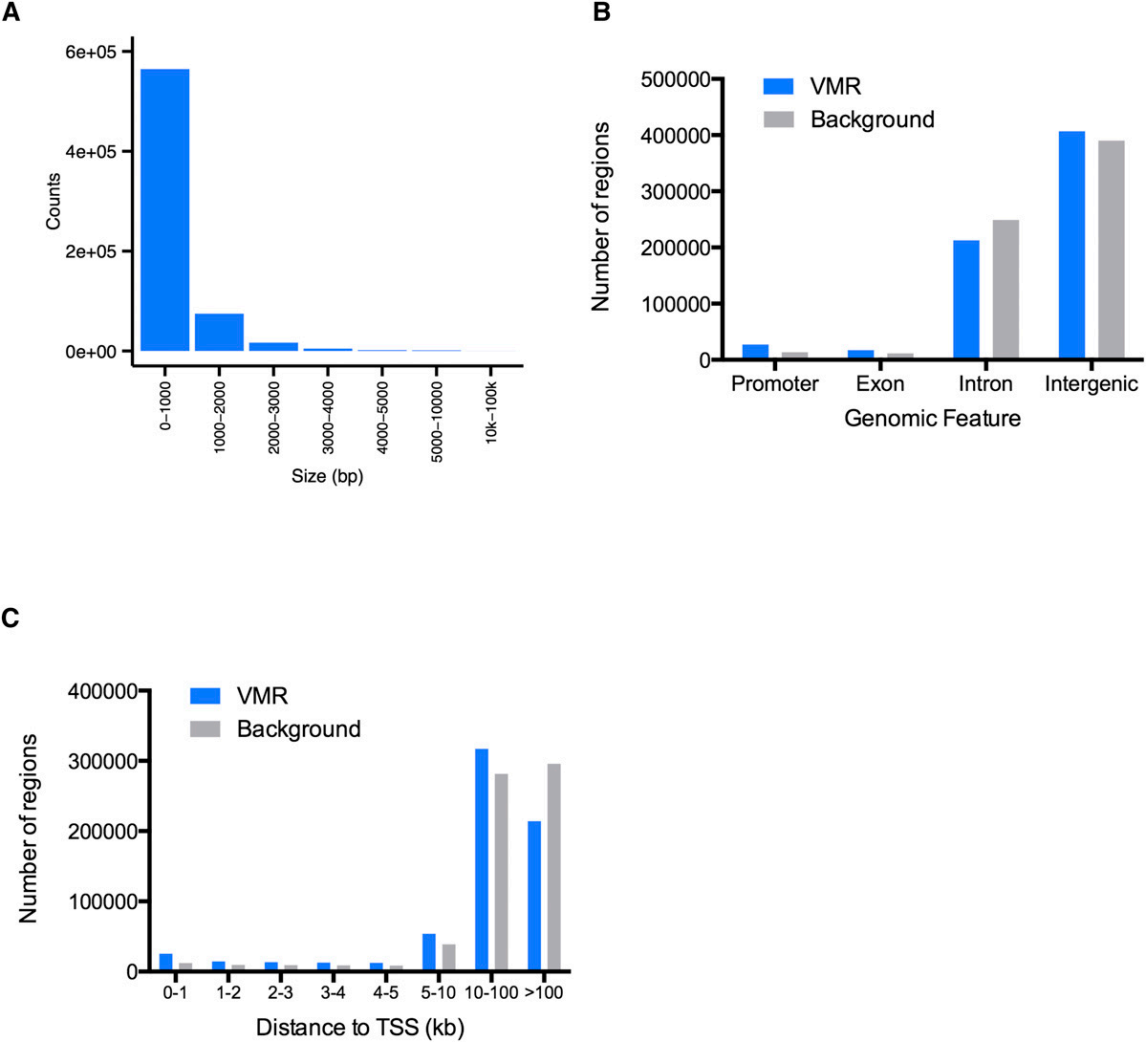
Majority of variably methylated regions (VMRs) are small and distal to transcription start sites (TSSs). (A) Distribution of VMR lengths at each range. (B) Distribution of VMRs and size matched randomly selected background regions in different genomic features. (C) Distance of VMRs and background regions to their nearest TSSs.

VMR lengths ranged from 2 bp (solo variably methylated CpG sites) up to 40 kb (Figure 2A and Figure S4A). The majority of the VMRs were short as 85% and 96% of VMRs were less than 1 kb and 2 kb in length, respectively. On average, each VMR contained nine CpGs (Figure S4B). The majority of the VMRs were located in introns and intergenic regions with only a small percentage overlapping promoters or exons, although they tended to overlap with promoters and exons more often than expected (Figure 2B).

### VMRs were distant from TSSs and enriched for TFBSs

Interestingly, the majority (88%) of VMRs were located more than 5 kb away from annotated transcription start sites (TSSs, Figure 2C), suggesting they might play a role as distal regulatory elements. Loss of DNA methylation in one germ layer was correlated with binding of lineage-relevant transcription factors during human ES cell differentiation (Tsankov *et al.* 2015). Indeed, hypomethylated regions in certain cells might give access to regulatory elements such as transcription factors or other DNA-binding proteins to modulate gene expression. To test this hypothesis in the cell types included in our study, we first determined the hypomethylated VMRs in each cell type (see *Materials and Methods*, Figure S4C). Then we integrated publicly available datasets from ENCODE to dissect the functional potential of these hypomethylated VMRs.

**Figure 3:**
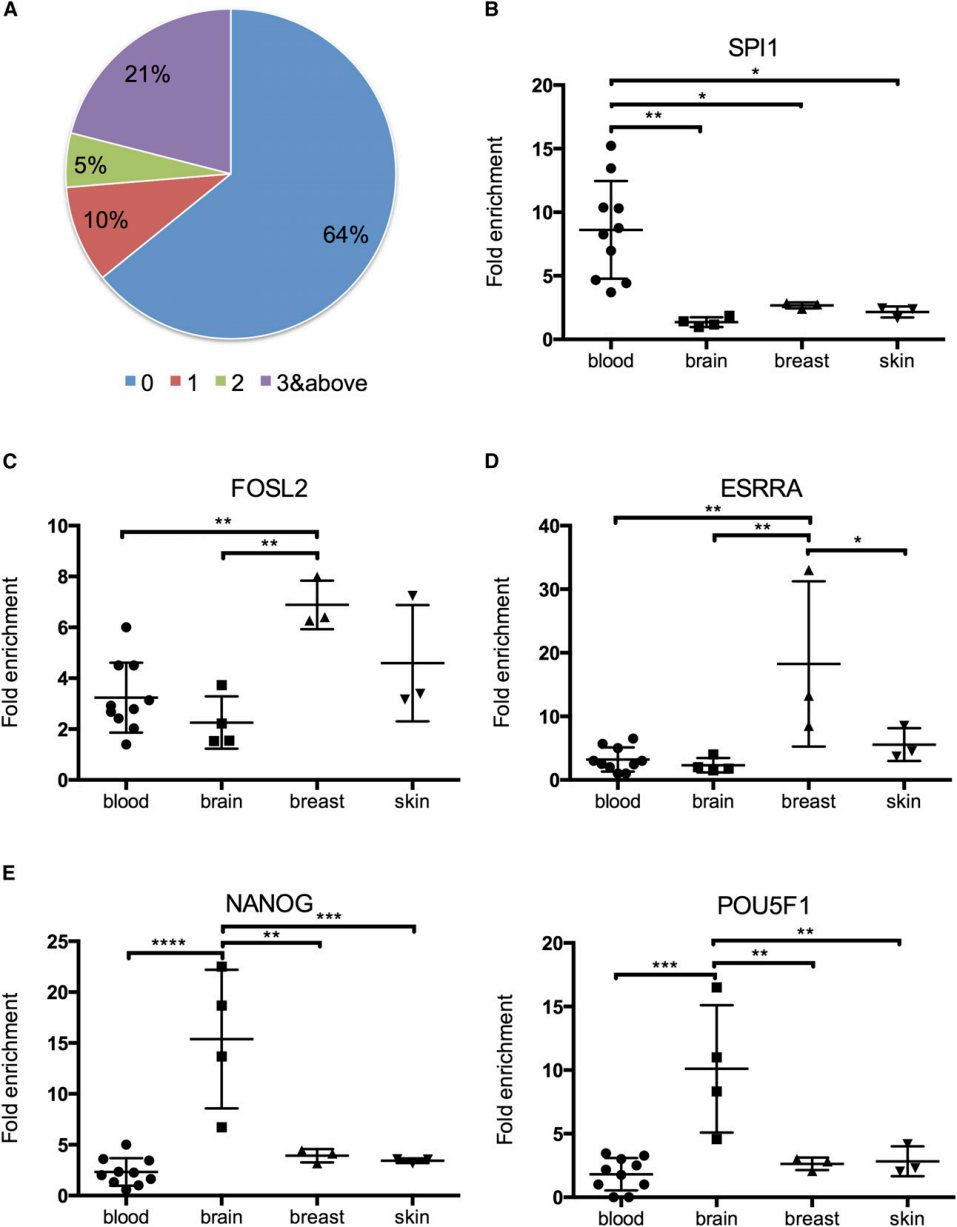
Variably methylated regions (VMRs) enrich for transcription factor binding sites in a tissue-specific manner. (A) Colocalization between all VMRs and transcription factor binding sites. Number of transcription factor binding peaks in each VMR were counted and the percentage of VMRs with 0, 1, 2, or 3 and more transcription factor binding peaks were calculated. Differential fold enrichment of functionally relevant transcription factor binding peaks in hypomethylated regions for (B) blood, (C) skin and breast, (D) breast, and (E) brain.

We first examined the colocalization between VMRs and TFBSs of more than 160 transcription factors defined by ChIP-seq peaks (The ENCODE Project Consortium 2012). A significant proportion of VMRs (36%) overlapped with at least one TFBS peak and this overlap was highly statistically significant (Figure 3A) (Fisher’s exact test, p-value < 2.2e-16). This evidence points to possible correlation between low DNA methylation and transcription factor binding that might be important for certain transcription factors to direct cellular transcriptional programs. In addition, 21% of VMRs overlapped with three or more TFBS peaks, suggesting potential interactions among different transcription factors.

To further pinpoint tissue type-specific contribution to these observed colocalizations of transcription factor binding and variable DNA methylation, we calculated the fold enrichment of TFBS peaks over size-matched randomly selected genomic regions and compared enrichment of transcription factor peaks among tissue types (See *Materials and Methods*). We found that the binding of many transcription factors exhibits tissue type-specific enrichment in hypomethylated VMRs (Figure 3, B–E). There are several interesting examples of transcription factors known to play important roles in particular tissue types. For example, FOSL2 is part of Activator protein 1 (AP-1), which regulates the expression of genes important in keratinocyte differentiation (Mehic *et al.* 2005). Its binding peaks were highly enriched in hypomethylated VMRs of skin (Figure 3C). Moreover, we found high enrichment of FOSL2 binding peaks located in VMRs hypomethylated in breast, which supports its role in breast cell development and carcinogenesis (Schröder *et al.* 2010). We also found ESRRA binding peaks highly enriched in hypomethylated VMRs of breast, where ESRRA is implicated in the etiology of breast cancer (Deblois *et al.* 2010). In addition, both NANOG and POU5F1 (OCT4) binding peaks were more highly enriched in hypomethylated VMRs of brain (Figure 3E). Both NANOG and POU5F1 are well-known master regulators of pluripotency; specifically, NANOG is implicated in both normal neural stem cell self-renewal and tumorigenesis in glioblastoma multiforme (GBM), while OCT4 functions in the differentiation of neural stem cells into neurons (Po *et al.* 2010; Zbinden *et al.* 2010; Deleidi *et al.* 2011).

In addition to the discovery of many transcription factor binding peaks enriched in hypomethylated VMRs, we also searched for transcription factor binding motifs in hypomethylated VMRs and found enrichment of motifs known to play important roles in each particular cell type (Figure S5). For example, LHX2 is a transcription factor important for neurogenesis in hippocampal development and its motif was found to be highly enriched in hypomethylated VMRs of various brain cell types (Subramanian *et al.* 2011). In many blood cell types, we found enrichment of motifs of ETS transcription factor family members such as ETS1 and ERG, which are well-known transcription factors involved in hematopoietic development (Zhang *et al.* 1995; Gallant and Gilkeson 2006). All the evidence supports potential interactions between variable DNA methylation and binding of transcription factors that direct cellular transcriptional machinery.

### VMRs colocalized with validated enhancers and enhancer histone modifications, and many possessed enhancer potential

**Figure 4:**
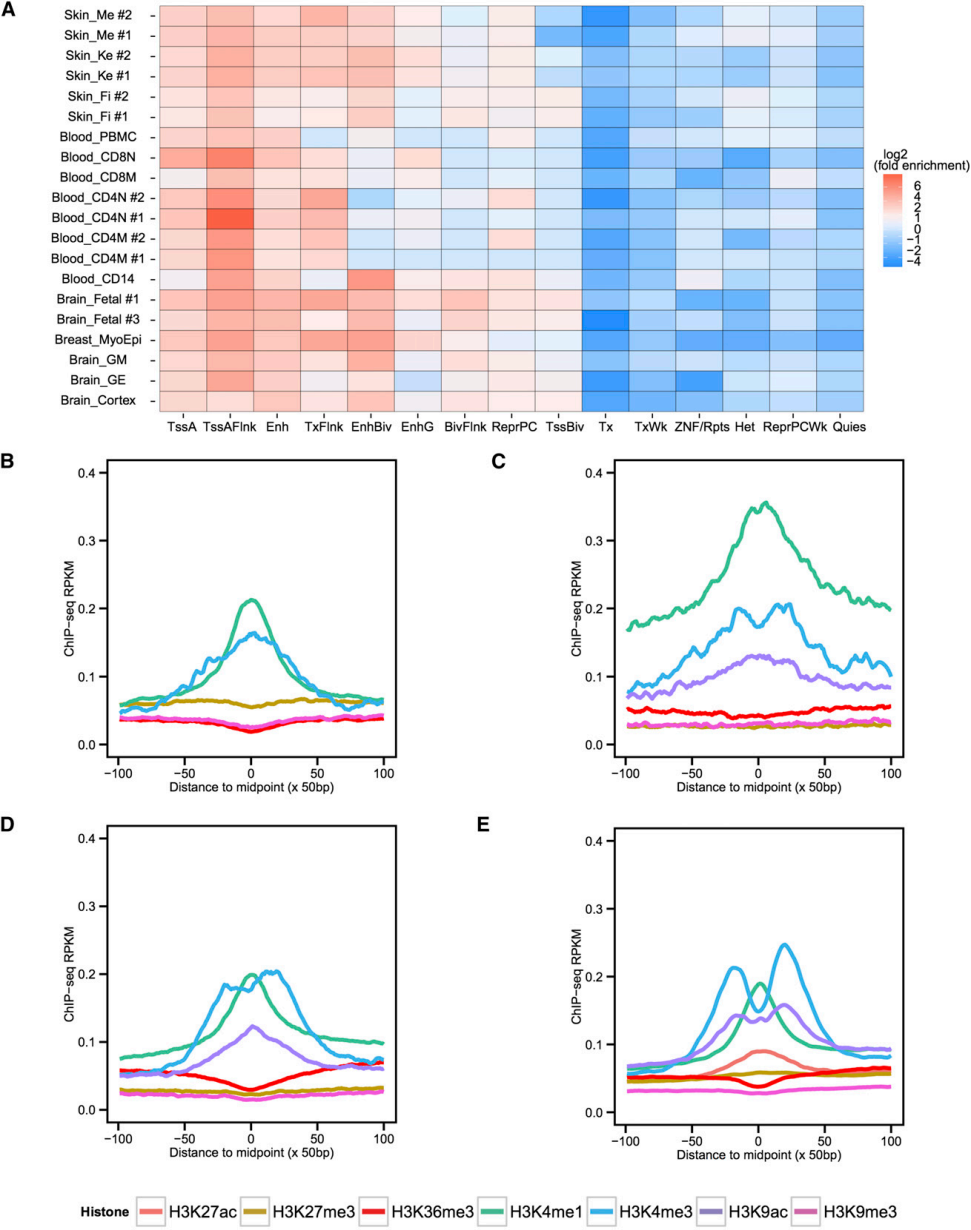
Variably methylated regions (VMRs) enrich for enhancer chromatin states and relevant histone modifications. (A) Enrichment of 15-state ChromHMM annotations in cell type-specific hypomethylated VMRs. Log2 transformed fold enrichment values for each ChromHMM state in each cell type were calculated and plotted. Replicate ChromHMM states were used for skin melanocytes, fibroblasts, keratinocytes, CD4 naïve cells, CD4 memory cells, and fetal brain, with replicate number indicated after cell type abbreviation detailed in Table S1. TssA, Active TSS; TssAFlnk, Flanking active TSS; Enh, Enhancers; TxFlnk, Transcribed at gene 5′ and 3′; EnhBiv, Bivalent enhancer; EnhG, Genic enhancers; BivFlnk, Flanking bivalent TSS/Enh; ReprPC, Repressed Polycomb; TssBiv, Bivalent/poised TSS; Tx, Strong transcription; TxWk, Weak transcription; ZNF/Rpts, ZNF genes + repeats; Het, Heterochromatin; ReprPCWk, Weak repressed Polycomb; Quies, Quiescent. ChIP-seq signal density RPKM values over a 10 kb region centered on hypomethylated VMRs for (B) cortex derived neurosphere cells, (C) CD8 naïve T cells, (D) breast myoepithelial cells, and (E) skin keratinocytes. RPKM values at 50 bp resolution were calculated and plotted.

Given the location of the identified VMRs being distal to TSSs, and the colocalization of transcription factor binding motifs and assay-identified binding sites, we sought to further explore their regulatory potential by examining their relationships with various histone modifications assayed on the same cells. We utilized available ChromHMM annotated chromatin states on each cell type and found enrichment of enhancer or active TSS-associated chromatin states in hypomethylated VMRs of the majority of cell types (Figure 4A) (Kundaje *et al.* 2015). In contrast, there was a depletion of heterochromatin and quiescent states in hypomethylated VMRs. We called ChIP-seq peaks of histone modifications for each cell type and found that up to 87% of the cell type-specific hypomethylated VMRs overlapped with enhancer histone modification ChIP-seq peaks represented by H3K4me1 in the same cell type (Figure S6A); the overlaps are statistically significant (Fisher’s exact test, p-value < 8e-10 for all). We also calculated the average signal density of ChIP-seq data over 10 kb regions centered around VMRs and found, in general, higher levels of enhancer or active transcription histone modification ChIP-seq signals centered in the VMRs compared to their flanking sequences (Figure 4, B–E, and Figure S6, B–E). These results led us to further hypothesize that many of the hypomethylated VMRs that we identified could possess potential enhancer activities in different cell types.

**Figure 5:**
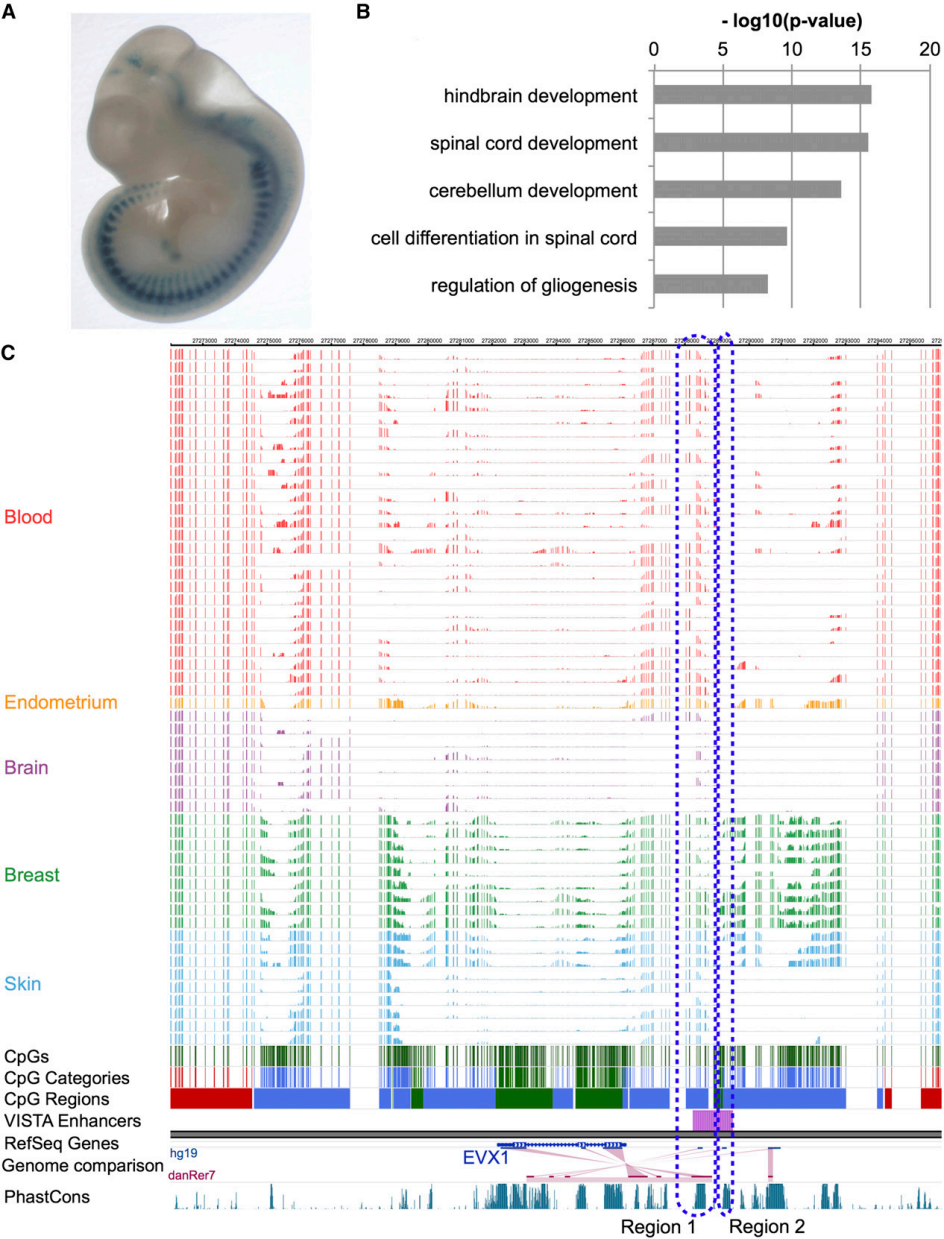
Genes near variably methylated regions (VMRs) enrich for relevant functions and certain VMRs were experimentally validated as enhancers. (A) VISTA enhancer element 629 was validated positive by transgenic mouse assay. (B) Gene Ontology term enrichment of genes near fetal brain hypomethylated VMRs. (C) WashU Epigenome Browser view of the methylation landscape around the EVX1 gene. VISTA enhancer element 629 overlaps with VMRs downstream of EVX1.

To test the enhancer hypothesis, we utilized the VISTA enhancer project’s validated enhancer data and determined whether our candidate VMRs could have enhancer activities (Visel *et al.* 2007). Indeed, we found that a significant portion (71%) of positively validated human VISTA enhancers overlapped with VMRs (Fisher’s exact test, p-value < 2.2e-16). As an example, VISTA enhancer hs629 was validated to have enhancer activity in mouse dorsal root ganglion and it overlapped with a VMR hypomethylated in fetal brain samples (Figure 5A). For a complete list of VMRs that overlap with positively validated VISTA enhancers, refer to Table S3.

To identify the genes and functions that could be potentially regulated by the hypomethylated VMRs, we analyzed the functional enrichment for genes near the VMRs in each cell type and found enrichment of genes whose functions were relevant for that particular cell type. For example, we found many genes near fetal brain-specific hypomethylated VMRs that encode functions such as hindbrain development and spinal cord development (Figure 5B, Figure S7, and Figure S8). We identified overlaps between several VMRs hypomethylated in brain and a positively validated VISTA enhancer (hs629) near the *EVX1* gene, which functions as a determinant of interneuron identity in spinal cord (Region 1 in Figure 5C). Interestingly, enhancer element hs629 contained a conserved noncoding element that was experimentally validated to have enhancer activities in interneurons in zebrafish, chick, and rat (Region 2 in Figure 5C) (Moran-Rivard *et al.* 2001; Suster *et al.* 2009). In different brain cell types, the winged-helix transcription factor FOXD3 has its promoter unmethylated in all the samples, but has multiple VMRs surrounding the gene and possibly regulating its expression and function specifically in brain. *FOXD3* is known to play important roles in establishing the neural crest lineage and in repressing melanogenesis in either mouse, chicken, or zebrafish (Kos *et al.* 2001; Stewart *et al.* 2006; Thomas and Erickson 2009). We found low methylation levels in brain and high methylation levels in skin melanocytes at these VMRs (Figure S9). Interestingly, three positively validated VISTA enhancers were located within a 460 kb region upstream of the *FOXD3* gene. In addition, BMP signaling, *PAX3*, and *PAX7* were also found near hypomethylated VMRs, and they are all part of the gene regulatory interaction networks important for neural crest development (Meulemans and Bronner-Fraser 2004). We found that another important neuronal transcription factor OLIG2 might be epigenetically regulated in brain (Figure S10) (Ligon *et al.* 2007). Several genes important in keratinocyte development and pathology were found to be linked to hypomethylation specifically in keratinocytes. For example, keratin 2 gene *KRT2* were found to be potentially regulated by hypomethylation in several VMRs upstream of *KRT2* (Figure S11) (Collin *et al.* 1992). In melanocytes, we found that *KIT*, *MITF*, *PAX3*, and *TYR* genes underlie functional enrichment, such as developmental pigmentation and melanocyte differentiation in melanocyte-specific hypomethylated VMRs. KIT and MITF are known to be important components of the melanocyte differentiation molecular machinery, with their individual activities and also functional interactions. MITF functions as a master regulator in melanocyte development and its regulation is mediated through transcription factor PAX3. One of its target genes is *TYR*, tyrosinase, which encodes the rate-limiting enzyme in melanin synthesis (Figure S12) (Hou *et al.* 2000; Levy *et al.* 2006; Yoshida *et al.* 2001; Mellgren and Johnson 2004; Kubic *et al.* 2008; Yang *et al.* 2008). All the evidence is consistent with the hypothesis that hypomethylated VMRs act as enhancers in specific cells to regulate nearby functionally related genes, possibly through transcriptional regulation mechanisms.

Lastly, to test whether cell type-specific hypomethylated VMRs could act as enhancers to mediate nearby gene expression, we analyzed gene expression data of the same samples to see whether we could observe expression differences correlated with methylation differences in VMRs. We identified genes near these VMRs, calculated their expression levels from RNA-seq data and compared them between two cell types. We found that between two cell types, genes near hypomethylated VMRs tend to have statistically significantly higher expression levels (Figure S13). This result supports our hypothesis that hypomethylated VMRs modulate nearby gene expression in specific cell types, further supporting their potential role as enhancers.

### VMRs enriched for GWAS variants

**Figure 6:**
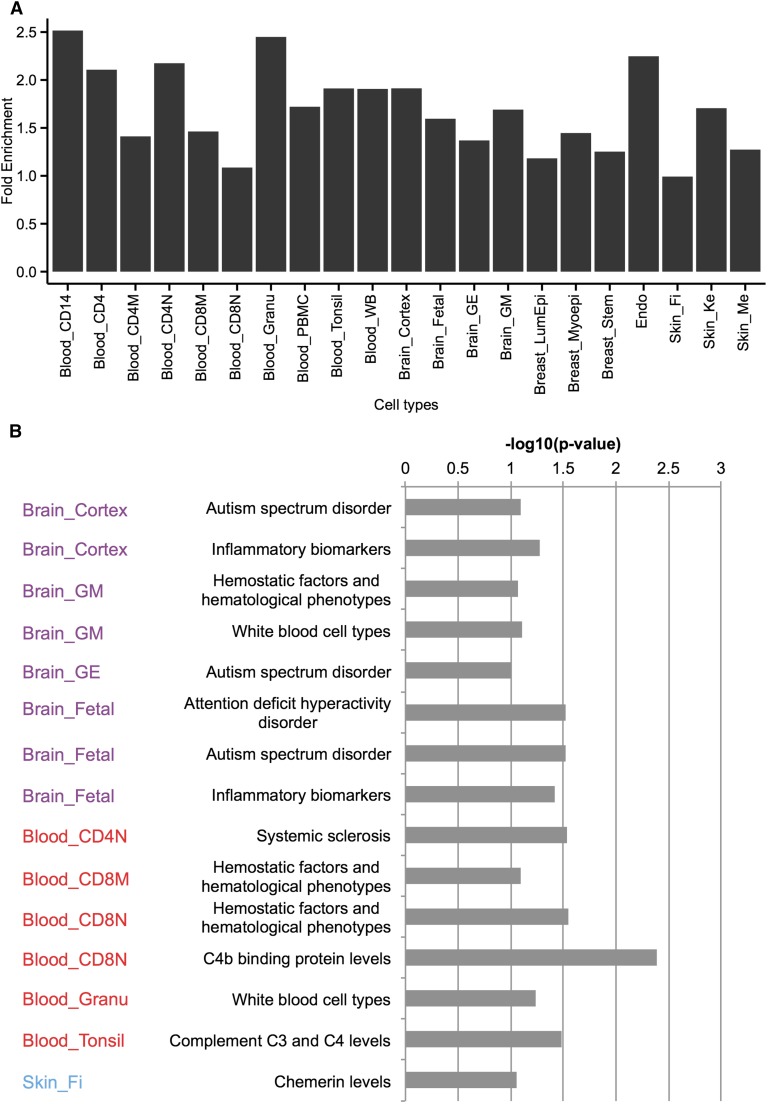
CpG methylation variation is linked to genetic variation and disease risk in different cells. (A) Fold enrichment of GWAS variants in hypomethylated VMRs of different cell types. (B) Examples of overrepresentations of functionally relevant GWAS traits in different cells. Hypergeometric p-values of significant enrichment of cell type-relevant GWAS traits in hypomethylated VMRs are shown.

GWAS have greatly aided our understanding of the genetic basis of many complex human traits and diseases. It was recently shown that variants identified from GWAS are often enriched in regulatory regions (The ENCODE Project Consortium 2012; Maurano *et al.* 2012). We hypothesized that VMRs may contain many genomic variants, whose functions could be regulated by epigenetic mechanisms such as DNA methylation. Thus, we examined the enrichment of genomic variants from many trait and disease-related GWAS within all possible hypomethylated VMRs defined in each cell type. We found a moderate but statistically significant association between VMRs and GWAS variants. Overall, VMRs encompassed 2197 published GWAS variants, representing a 1.4-fold enrichment (Fisher’s exact test, p-value < 2.2e-16), and enrichment of GWAS variants was higher in certain cell types (Figure 6A). 96.4% of the GWAS variants were located in noncoding regions highlighting the regulatory potential of noncoding sequences. Furthermore, 94.3% of GWAS variants located within VMRs were noncoding. By interrogating the relationship between GWAS and VMRs, the DNA methylation levels of VMRs provide an epigenetic annotation of many GWAS variants, which might be useful in understanding the functional relevance of a GWAS variant in a cell type (Zhou *et al.* 2015). We calculated the enrichment of GWAS traits linked to variants in hypomethylated VMRs and found enrichment of GWAS traits in relevant cell types (Figure 6B). There was significant enrichment of neuronal disease-related traits in different brain cell types. Interestingly, some immunological function-related traits were enriched in VMRs hypomethylated in brain, in agreement with recent findings concerning the link between the dysfunction of the immune system and Alzheimer’s disease (Heneka *et al.* 2015; Matsuda *et al.* 2015; Gjoneska *et al.* 2015; Chakrabarty *et al.* 2015; Guillot-Sestier *et al.* 2015). When we looked at individual variants, we found variants associated with red blood cell traits, white blood cell count, and IgG glycosylation within blood hypomethylated VMRs; variants associated with Alzheimer’s disease, schizophrenia, and multiple sclerosis within brain-related VMRs; and variants associated with systemic sclerosis and psoriasis within skin-related VMRs. This limited evidence could be taken to suggest that variants not only tag the disease but may also be within regulatory sites through which DNA methylation may influence phenotypes or disease susceptibility. For a complete list of all the GWAS variants located in hypomethylated VMRs, refer to Table S4.

To further investigate whether DNA methylation could play a role in the regulation of these variants, we focused on variants that colocalized with variably methylated CpGs. Among the 2197 GWAS variants within VMRs, 30 were in a CpG context. Since DNA methylation levels of VMRs can be regulatory, the variants could potentially have a direct impact on gene regulation by influencing DNA methylation of the CpGs underlying the variants, providing a mechanistic link between genetic variants and their associated traits or diseases. For example, rs10499197 was found to be a susceptibility locus for systemic lupus erythematosus and this CpG was hypomethylated in several blood cell types. The methylation level of CpGs and VMRs might thus influence the risk of disease. For a complete list of all the GWAS variants that in a CpG context, refer to Table S5.

### Comparison with WGBS-based dynamic CpGs and regions

Ziller *et al.* (2013) published a seminal paper detailing 42 DNA methylomes using WGBS in normal developmental tissues and cultured cell lines. The major conclusion from this work was that 21.8% of autosomal CpGs showed dynamic methylation levels that colocalized with enhancer and other gene regulatory elements. Similarly, Schultz *et al.* (2015) recently profiled 18 tissue types and found 15.4% of CpGs to be differentially methylated. Since our study employed different technologies and profiled many nonoverlapping sample types, we determined the extent to which their differentially methylated regions (DMRs) overlap with our VMRs.

To test the similarity between the methylation levels of our methylCRF prediction and those of WGBS profiled on the same cell types, we calculated their genome-wide concordance (defined as the percentage of CpGs with a methylation level difference less than 25%) between samples profiled by both Ziller *et al.* (2013) and this study (See *Materials and Methods*). Although the samples were from different sources, the concordance between tested pairs was as high as 85.3%, and methylCRF predictions of CpG methylation levels were comparable to WGBS (Table S6). This is consistent with previously reported results (Stevens *et al.* 2013; Lee *et al.* 2015).

We compared the overlap of variably methylated CpGs between ours and those defined in Ziller *et al.* (2013) and found that, although their numbers are similar, 36.1% of variably methylated CpGs identified by methylCRF data only account for 38.4% of variably methylated CpGs identified by their WGBS data (Figure S14A). The number of VMRs was smaller than that of DMRs (663,916 *vs.* 716,087), and 40.4% of VMRs overlapped with 26.8% of DMRs defined in Ziller *et al.* (2013) (Figure S14B). Similarly, 58.2% of VMRs overlapped with 29.9% of DMRs defined in Schultz *et al.* (2015) (Figure S14B). Even two sets of DMRs defined by two WGBS studies showed small overlaps (Figure S14B). The WGBS DMRs covered more genome sequences than those of VMRs (315 million bases for VMRs *vs.* 492 million bases for Ziller DMRs *vs.* 384 million bases for Schultz DMRs) (Figure S14C). When we looked at the set of regions defined in all 3 studies, only up to 27.1% of regions were shared among three studies [27.1% of VMRs, 21.3% of Ziller *et al.* (2013) DMRs, and 14.3% of Schultz *et al.* (2015) DMRs] (Figure S14D). The overlap between VMRs and DMRs was small in scale, suggesting that the analysis of dynamic DNA methylation has not yet reached saturation, as also suggested in Schultz *et al.* (2015). In addition to profiling different samples, differences in DNA methylation profiling technology, analysis methods, as well as statistical cutoffs employed by different studies (the present study; Schultz *et al.* 2015; Ziller *et al.* 2013), could have also contributed to our observation that the identification of dynamic DNA methylation has not reached saturation. One reason for the genome coverage difference comes from the fact that Ziller *et al.* (2013) extended single CpG DMRs to 100 bp. 36.6% of DMRs were derived from a single CpG whereas 19.2% of VMRs were single CpG VMRs (Figure S14E). This difference could be due to the fact that WGBS relies on a sufficient number of reads to accurately estimate methylation levels, and some of the single CpG DMRs could have low coverage and thus lead to inaccurate methylation calls. Indeed, we found that single CpG DMRs from Ziller *et al.* (2013) had significantly lower read coverage than the rest of the CpGs covered by DMRs (Figure S14F, Wilcoxon rank-sum test, p-value < 0.05). If we only considered the nonsingle-CpG regions in both sets, we observed almost the same percentage of covered base overlap and a slightly higher overlap of regions between VMRs and DMRs (Figure S14, G–H). The discrepancy, not explained by single-CpG regions, is probably due to different cell types profiled in the two studies as only a few cell types were profiled in both, and also the inherent difference between WGBS and MeDIP-seq and MRE-seq methods used to define them or both.

By comparing regions identified uniquely in each study, we found that they shared similar size and genomic distribution (Figure S15, A–D). VMR-specific regions had overall higher overlap with promoters and intergenic regions, and lower overlap with intronic regions (Figure S15D). When we examined the colocalization with TFBSs, the distribution was very similar across study-specific regions (Figure S15, E–G) although they all showed a decrease in the proportion of VMRs harboring potential TFBSs compared to the complete VMR set (Figure 3A).

### Reference methylation dynamics browser track

Using our segmentation, we created a DNA methylation dynamics track (CpG Regions Extended) for visualization and reference on the Washington University Epigenome Browser (http://epigenomegateway.wustl.edu/browser/ or Visualization on the companion website, see *Materials and Methods*). It provides a summarized source of insight about the potential activity of genomic regions of interest. This resource should facilitate the interpretation of and hypothesis generation about genomic and epigenomic data.

## Discussion

In this study, we applied methylCRF to a total of 54 samples spanning multiple cell and tissue types. We categorized CpGs based on their DNA methylation patterns across all the samples examined. A longstanding observation is that the majority of CpGs in the genome are methylated. This was confirmed in our analysis, where ∼70% of the autosomal CpGs were constitutively methylated (≥ 70% CpG methylation) regardless of the cell type. In contrast, 7.5% of the autosomal CpGs were constitutively unmethylated (≤ 30% CpG methylation) and, as we have shown, most of these unmethylated regions are located in CpG islands. In addition, the majority of the constitutively unmethylated regions (UMRs) are less than 2 kb. Compared to VMRs, UMRs are much more enriched in regions close to TSSs, with over a third of them located in promoter regions (Figure S16).

We identified 22.6% of the genomic CpGs that show variable DNA methylation across 54 samples. These CpGs could be functional in different cell types by varying their methylation levels to influence the transcriptional network in a particular cell type. We combined CpGs into windows and characterized them by looking at their size, distribution, and relationship with various TFBSs, motifs, histone modifications, enhancer potentials, and GWAS variants. We have compelling evidence to show that many of the VMRs are distal to known TSSs and possess enhancer or active transcription histone modifications. They not only harbor many known transcription factor binding peaks but also many functionally relevant transcription factor binding motifs. Many of these regions have been experimentally validated. With the availability of more experimental data, more regions are expected to have enhancer activities in certain cell or tissue types. We found that a considerable number of GWAS variants overlap with variably methylated CpGs, suggesting a possible mechanism for the regulation or dis-regulation of these trait or disease-associated variants.

Our results suggest widespread colocalization between transcription factor binding and identified VMRs, even given the currently limited amount of transcription factor ChIP-seq data. It is tempting to postulate that those VMRs that did not overlap with current transcription factor binding events might also harbor regulatory potentials through the modulation of transcription factor binding.

One striking feature we uncovered is that, regardless of the cell type, roughly the same percentage of CpGs are being methylated and unmethylated, yet exactly which CpGs in a particular cell type are unmethylated is cell type-dependent (Figure 1A). This discovery suggests that a cell is able to maintain its epigenome at a default level. In another words, roughly 11% of the CpGs will be unmethylated in any given cell type and the epigenome somehow directs unmethylation in CpGs that are important in cell type specification. It would be very interesting to understand the molecular basis and mechanisms of this process in detail.

We have previously shown that methylCRF is highly robust in predicting single-CpG methylation levels genome-wide with high accuracy in comparison to WGBS but at a much lower cost (Stevens *et al.* 2013). Here, we present the first multiple cell type DNA methylome analysis using methylCRF predictions. Our study again demonstrates the power of combining two complimentary techniques for measuring genome-wide DNA methylation and statistically learning single-CpG-resolution DNA methylation levels in multiple different cell types. In addition, we were able to leverage methylCRF to identify biologically important regions with variable DNA methylation. Based on our analysis, we speculate that more variably methylated CpGs will be identified as more cell types are sequenced and analyzed (Figure S17). We would argue that the overall findings are consistent with those derived from WGBS data and that the results clearly suggest that DNA methylation plays a role in the regulation of cell or tissue type-specific gene functions through distal enhancer sites. They highlight the plausible link between genetic variation and epigenetic regulation. The data generated and results presented here will be of great interest to the research community. Our methylation predictions in many cell types could be utilized to complement other studies in deciphering the epigenomic landscape of cells. The genome segmentation data and track could be very useful in bringing the analytical focus on certain important regions in different studies. The combined use of MeDIP-seq and MRE-seq through methylCRF continues to be an economical yet powerful method to generate complete DNA methylomes. Using the library coverage statistics in the WGBS and our study, we estimate that a WGBS methylome costs more than 10 times that of a combined MeDIP-seq and MRE-seq methylome (Table S7). Our methods will greatly facilitate the application of methods utilizing DNA methylome data along with other genomic data, such as predicting TFBSs from DNA methylation data (Xu *et al.* 2015). With our data and the availability of many more single-CpG-resolution DNA methylome data in the foreseeable future, we expect the field will witness greater insights gained from integrating data from a complete cell type collection.

## 

## Supplementary Material

Supplemental Material
