# Supplementary material for: Mapping of Variable DNA Methylation Across Multiple Cell Types Defines a Dynamic Regulatory Landscape of the Human Genome
Source: G3 (Bethesda). 2016 Feb 16;6(4):973–86. doi: 10.1534/g3.115.025437 (PMC4825665; doi:10.1534/g3.115.025437)
Supplement: Supplemental Material [file supp_6_4_973__index.html]

Mapping of Variable DNA Methylation across Multiple Cell Types Defines a Dynamic Regulatory Landscape of the Human Genome — Mapping of Variable DNA Methylation Across Multiple Cell Types Defines a Dynamic Regulatory Landscape of the Human Genome — Supplemental Material 

# Mapping of Variable DNA Methylation Across Multiple Cell Types Defines a Dynamic Regulatory Landscape of the Human Genome

## Supplemental Material for Gu *et al*., 2016

**Files in this Data Supplement:**

- Figure S1 - Characterization of autosomal CpG methylation patterns continued. (.pdf, 242 KB)
- Figure S2 - Overlap of constitutively methylated CpGs with repeats and constitutively unmethylated CpGs with CpG islands. (.pdf, 74 KB)
- Figure S3 - A complete browser view of the example in Figure 1D. (.pdf, 255 KB)
- Figure S4 - Characterization of variably methylated regions (VMRs) continued. (.pdf, 90 KB)
- Figure S5 - Known transcription factor binding motifs enriched in VMRs for each tissue type and their enrichment in (A) brain (B) blood (C) skin and (D) breast. (.pdf, 79 KB)
- Figure S6 - Hypomethylated VMRs enrich for enhancer or active transcription histone modifications. (.pdf, 134 KB)
- Figure S7 - Hierarchy of functional enrichment of genes near hypomethtlated VMRs in fetal brain. (.pdf, 517 KB)
- Figure S8 - Functional enrichment of genes near hypomethylated VMRs. (.pdf, 92 KB)
- Figure S9 - *FOXD3* is potentially regulated by methylation level in nearby regions in brain. (.pdf, 1,302 KB)
- Figure S10 - *OLIG2* is potentially regulated by upstream VMRs in fetal brain. (.pdf, 1,040 KB)
- Figure S11 - *KRT2* is potentially regulated by upstream VMRs in keratinocyte. (.pdf, 193 KB)
- Figure S12 - *TYR* is potentially regulated by VMRs in melanocyte. (.pdf, 319 KB)
- Figure S13 - Genes near hypomethylated VMRs show increased expression. (.pdf, 98 KB)
- Figure S14 - Comparison between methylCRF predicted VMRs and WGBS predicted DMRs. (.pdf, 2,082 KB)
- Figure S15 - Characterization of regions identified specifically in each study. (.pdf, 578 KB)
- Figure S16 - Characterization of constitutively unmethylated regions (UMRs). (.pdf, 364 KB)
- Figure S17 - Saturation analysis on the identification of variably methylated CpGs. (.pdf, 456 KB)
- Table S1 - Data used in this study. (.xlsx, 35 KB)
- Table S2 - CpG category number and percentage in the genome. (.xlsx, 43 KB)
- Table S3 - List of VMRs overlapping positive human VISTA. (.xlsx 87 KB)
- Table S4 - GWAS variants located in VMRs by cell type. (.xlsx, 192 KB)
- Table S5 - GWAS variants in CpG context. (.xlsx, 28 KB)
- Table S6 - Concordance between WGBS and methylCRF. (.xlsx, 9 KB)
- Table S7 - Bases sequenced comparison between WGBS and methylCRF. (.xlsx, 35 KB)
