# Supplementary material for: Mapping of Variable DNA Methylation Across Multiple Cell Types Defines a Dynamic Regulatory Landscape of the Human Genome
Source: G3 (Bethesda). 2016 Feb 16;6(4):973–86. doi: 10.1534/g3.115.025437 (PMC4825665; doi:10.1534/g3.115.025437)
Supplement: Supplemental Material [file supp_g3.115.025437_FigureS1.pdf]

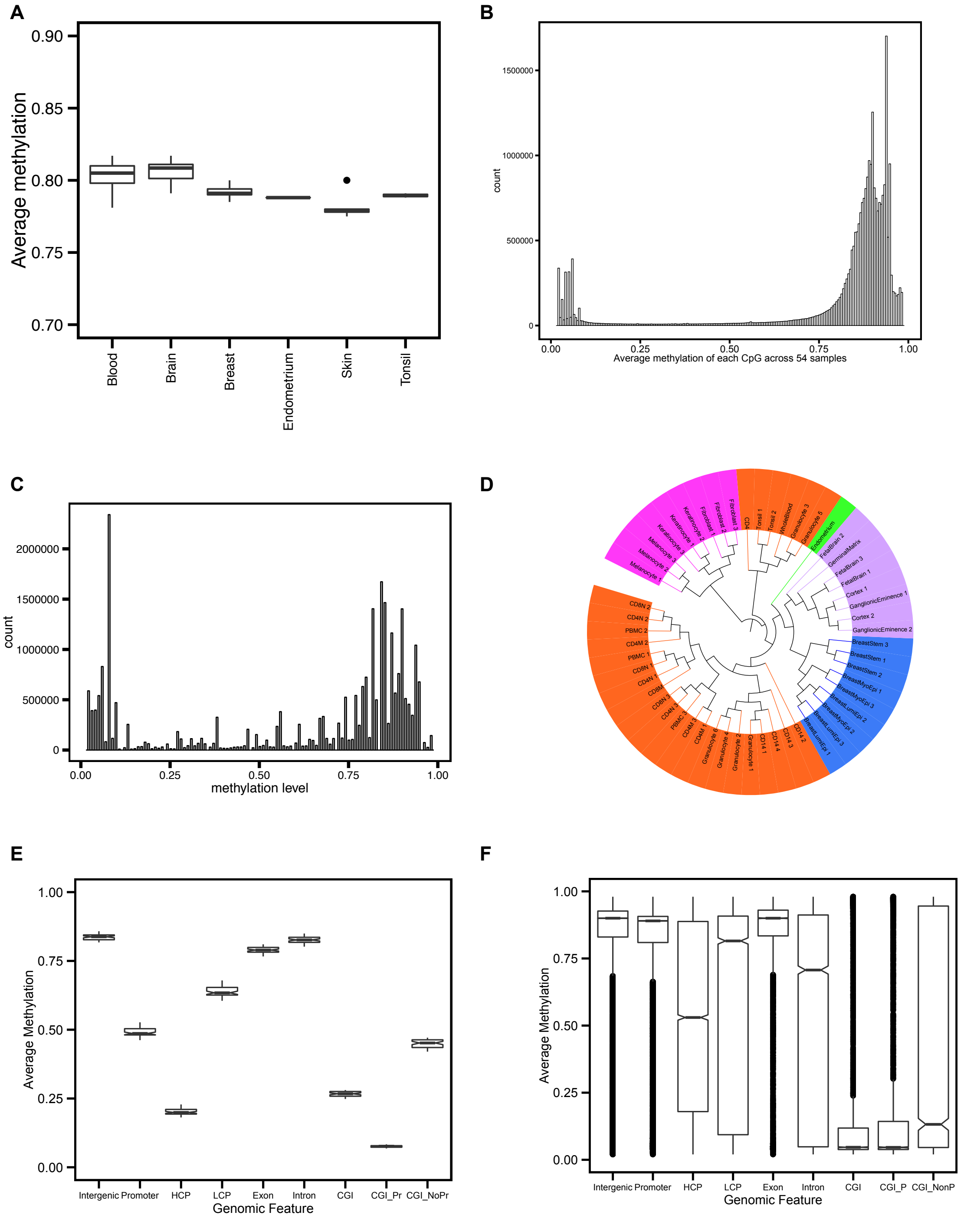
A
Average methylation
0.90
0.85
0.80
0.75
0.70
Blood
Brain
Breast
Endometrium
Skin
Tonsil
B
count
1500000
1000000
500000
0
0.00
0.25
0.50
0.75
1.00
Average methylation of each CpG across 54 samples
C
count
2000000
1500000
1000000
500000
0
0.00
0.25
0.50
0.75
1.00
methylation level
D
E
Average Methylation
1.00
0.75
0.50
0.25
0.00
Intergenic Promoter HCP LCP Exon Intron CGI CGI_Pr CGI_NoPr
Genomic Feature
F
Average Methylation
1.00
0.75
0.50
0.25
0.00
Intergenic Promoter HCP LCP Exon Intron CGI CGI_P CGI_NonP
Genomic Feature

Figure S1. Characterization of autosomal CpG methylation patterns continued.

A. Boxplot of average genome-wide single CpG methylation levels for different cell types of the same tissue type.
B. Genome-wide distribution of average methylation levels for each CpG across 54 samples.
C. Frequency plot of the lowest DNA methylation levels across all the samples for all the CpGs.
D. Clustering of 54 samples based on average methylation levels at 1kb sliding windows. Average methylation levels of all the 1kb windows were used as input in hierarchical clustering and the resulting clustering were visualized using iTOL (Letunic and Bork 2007).
E. Mean methylation levels of CpGs in different genomic features across 54 samples. Promoter: 2kb regions upstream of transcription start sites; HCP: High CpG Promoters; LCP: Low CpG Promoters; CGI: CpG Islands; CGI_Pr: CpG Islands overlapping with promoters. CGI_NoPr: CpG Islands not overlapping with promoters.
F. A representative boxplot showing distribution of average methylation levels of CpGs in various genomic features for the #3 skin melanocyte sample.
