# Supplementary material for: Mapping of Variable DNA Methylation Across Multiple Cell Types Defines a Dynamic Regulatory Landscape of the Human Genome
Source: G3 (Bethesda). 2016 Feb 16;6(4):973–86. doi: 10.1534/g3.115.025437 (PMC4825665; doi:10.1534/g3.115.025437)
Supplement: Supplemental Material [file supp_g3.115.025437_FigureS2.pdf]

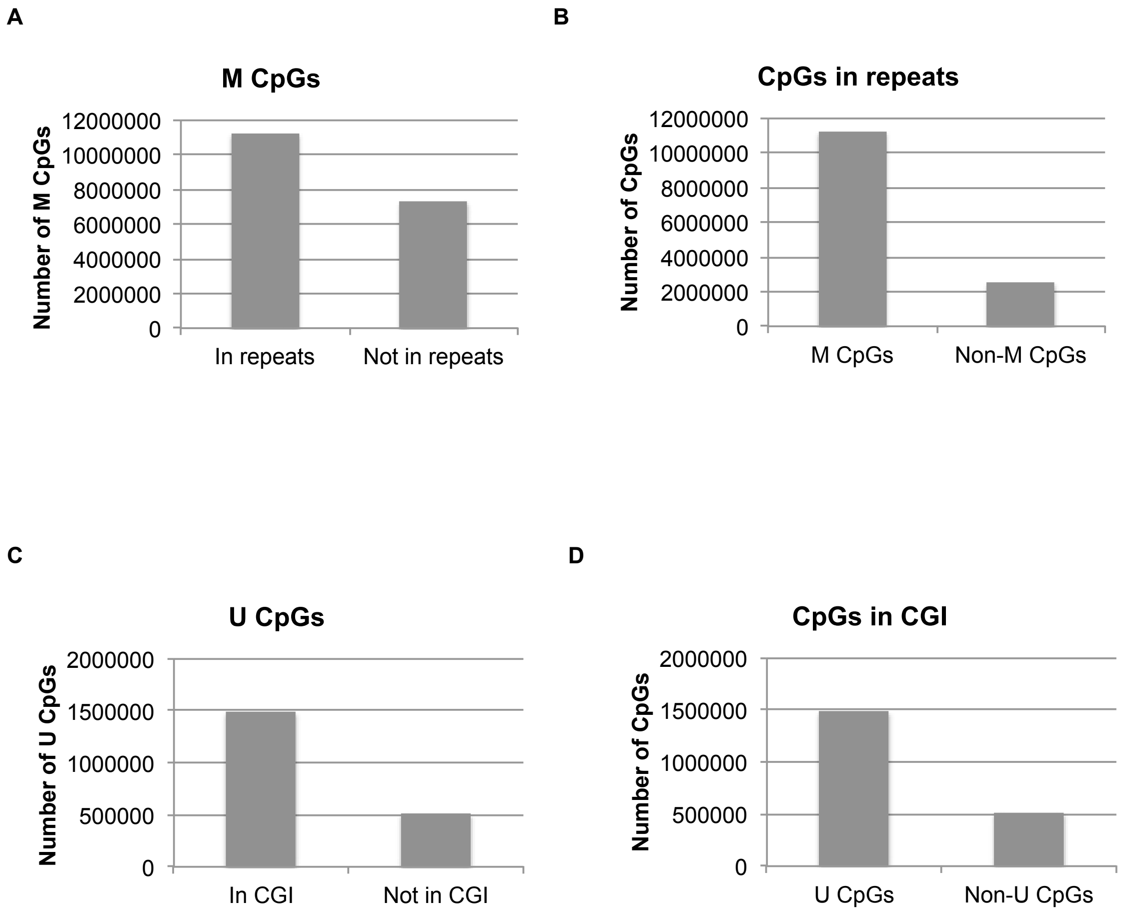


Figure S2. Overlap of constitutively methylated CpGs with repeats and constitutively unmethylated CpGs with CpG islands.

A. Number of constitutively methylated CpGs (M CpGs) in repeats vs. not in repeats.
B. Number of constitutively methylated CpGs (M CpGs) and non-constitutively methylated CpGs (Non-M CpGs) in repeats.
C. Number of constitutively unmethylated CpGs (U CpGs) in CpG islands (CGI) vs. not in CpG islands.
D. Number of constitutively unmethylated CpGs (U CpGs) and non-constitutively unmethylated CpGs (Non-U CpGs) in CpG islands.
