# Supplementary material for: Mapping of Variable DNA Methylation Across Multiple Cell Types Defines a Dynamic Regulatory Landscape of the Human Genome
Source: G3 (Bethesda). 2016 Feb 16;6(4):973–86. doi: 10.1534/g3.115.025437 (PMC4825665; doi:10.1534/g3.115.025437)
Supplement: Supplemental Material [file supp_g3.115.025437_FigureS3.pdf]

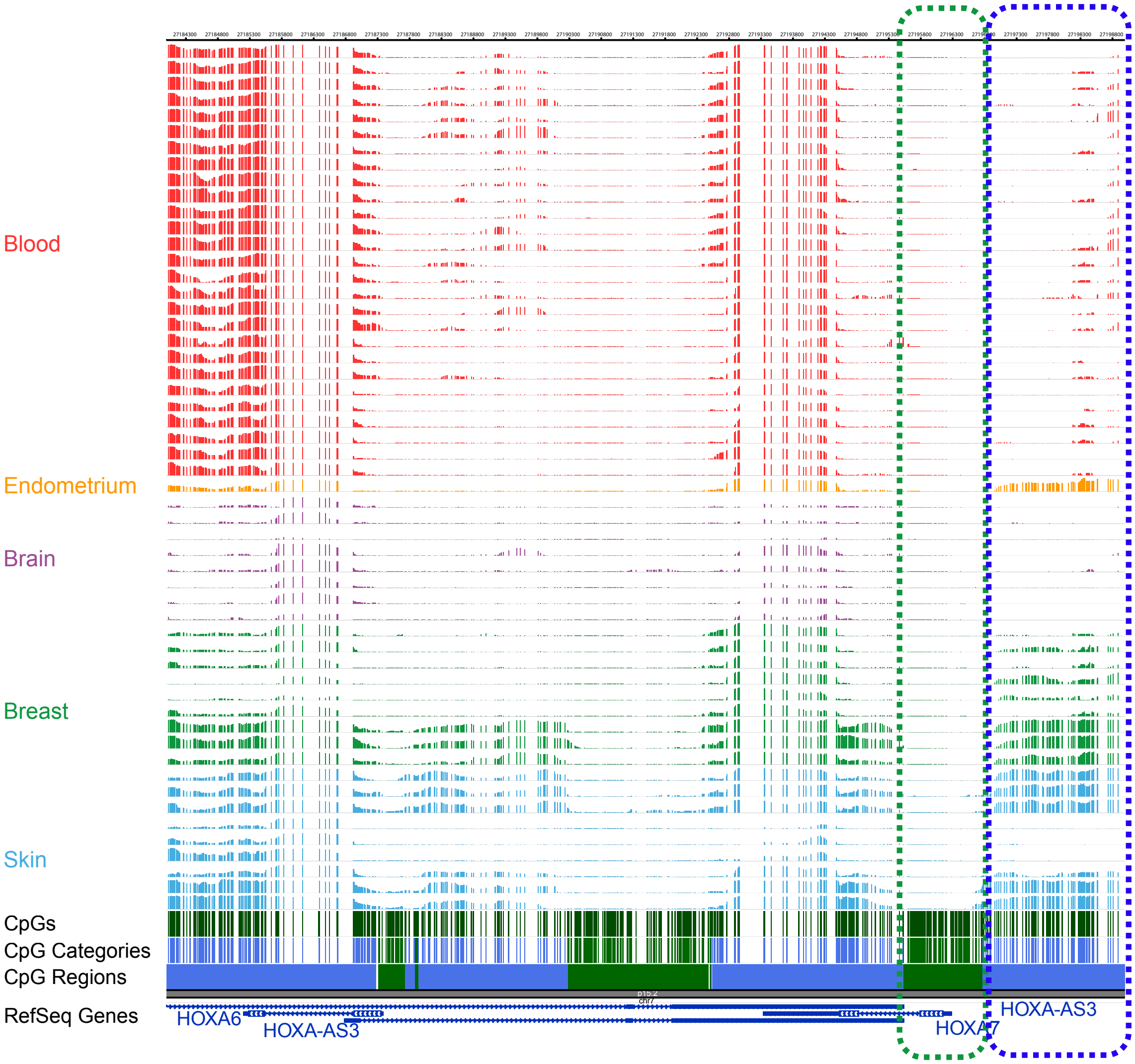

Figure S3. A complete browser view of the example in Figure 1D.

Shown is the proximal methylation landscape around *HOXA7*. *HOXA7* was shown to play a role in repressing genes associated with keratinocyte differentiation (La Celle 2001). The browser shot shows specific low methylation near *HOXA7* gene in keratinocyte and high level of methylation in other skin cell types. The order of 54 methylCRF tracks follows the ID numbers listed in Supplemental Table S1 for all the complete browser views.
