# Supplementary material for: Mapping of Variable DNA Methylation Across Multiple Cell Types Defines a Dynamic Regulatory Landscape of the Human Genome
Source: G3 (Bethesda). 2016 Feb 16;6(4):973–86. doi: 10.1534/g3.115.025437 (PMC4825665; doi:10.1534/g3.115.025437)
Supplement: Supplemental Material [file supp_g3.115.025437_FigureS4.pdf]

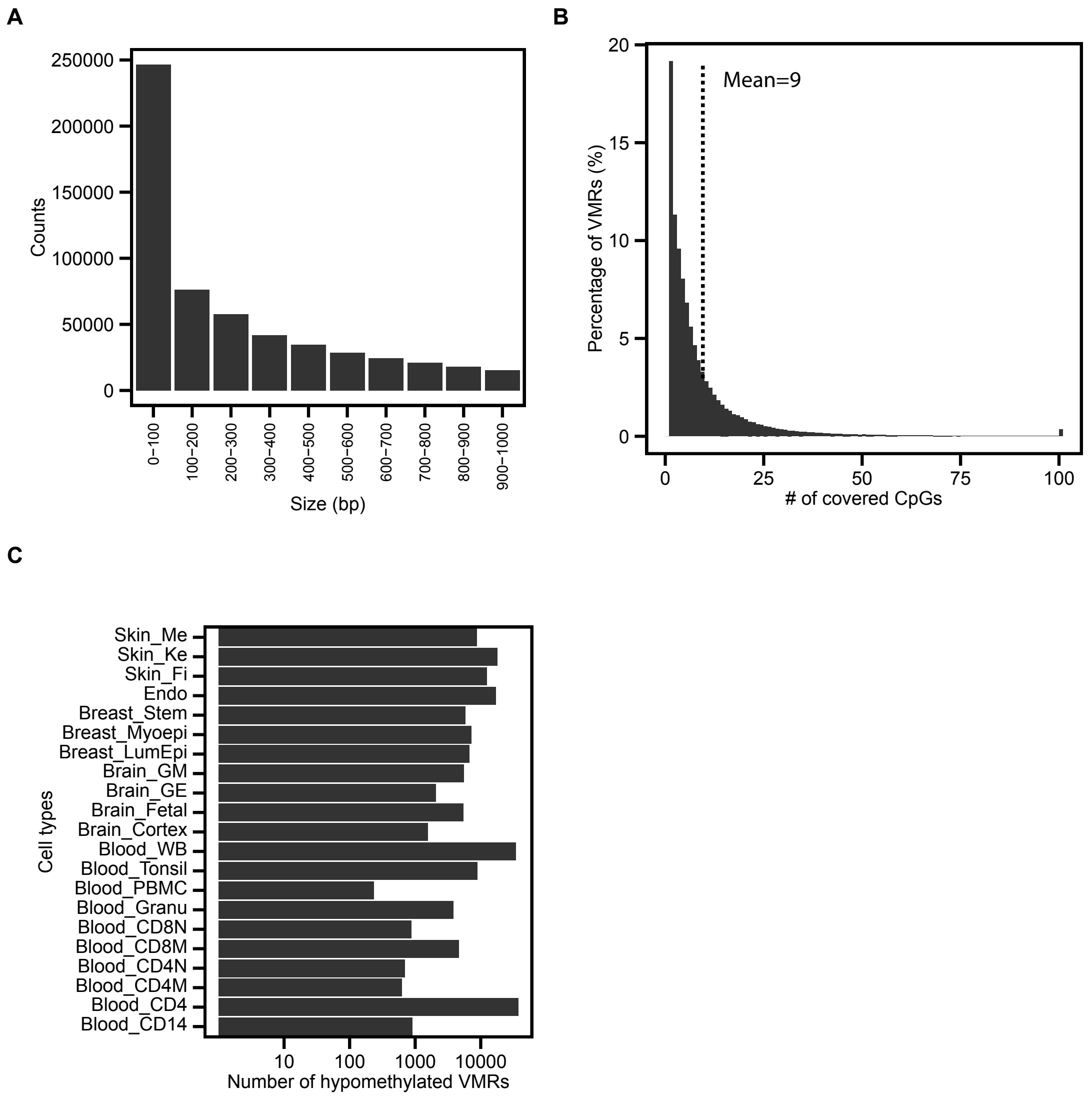

Figure S4. Characterization of variably methylated regions (VMRs) continued.

A. Histogram of sizes of VMRs within 0-1000 bp range.

B. Histogram of the number of CpGs covered in VMRs.

C. Number of hypomethylated VMRs in each cell type.
