# Supplementary material for: Mapping of Variable DNA Methylation Across Multiple Cell Types Defines a Dynamic Regulatory Landscape of the Human Genome
Source: G3 (Bethesda). 2016 Feb 16;6(4):973–86. doi: 10.1534/g3.115.025437 (PMC4825665; doi:10.1534/g3.115.025437)
Supplement: Supplemental Material [file supp_g3.115.025437_FigureS5.pdf]

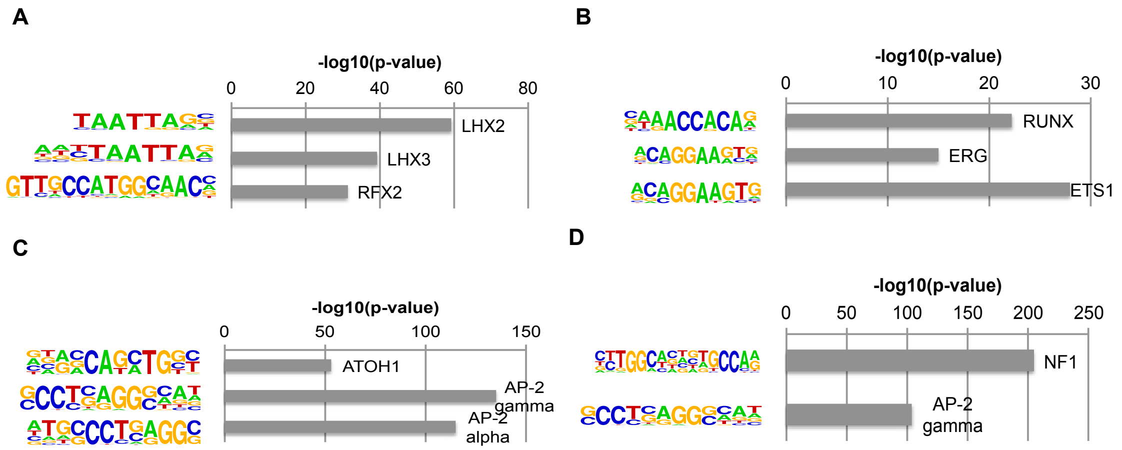

Figure S5. Known transcription factor binding motifs enriched in VMRs for each tissue type and their enrichment in (A) brain (B) blood (C) skin and (D) breast.
