# Supplementary material for: Mapping of Variable DNA Methylation Across Multiple Cell Types Defines a Dynamic Regulatory Landscape of the Human Genome
Source: G3 (Bethesda). 2016 Feb 16;6(4):973–86. doi: 10.1534/g3.115.025437 (PMC4825665; doi:10.1534/g3.115.025437)
Supplement: Supplemental Material [file supp_g3.115.025437_FigureS6.pdf]

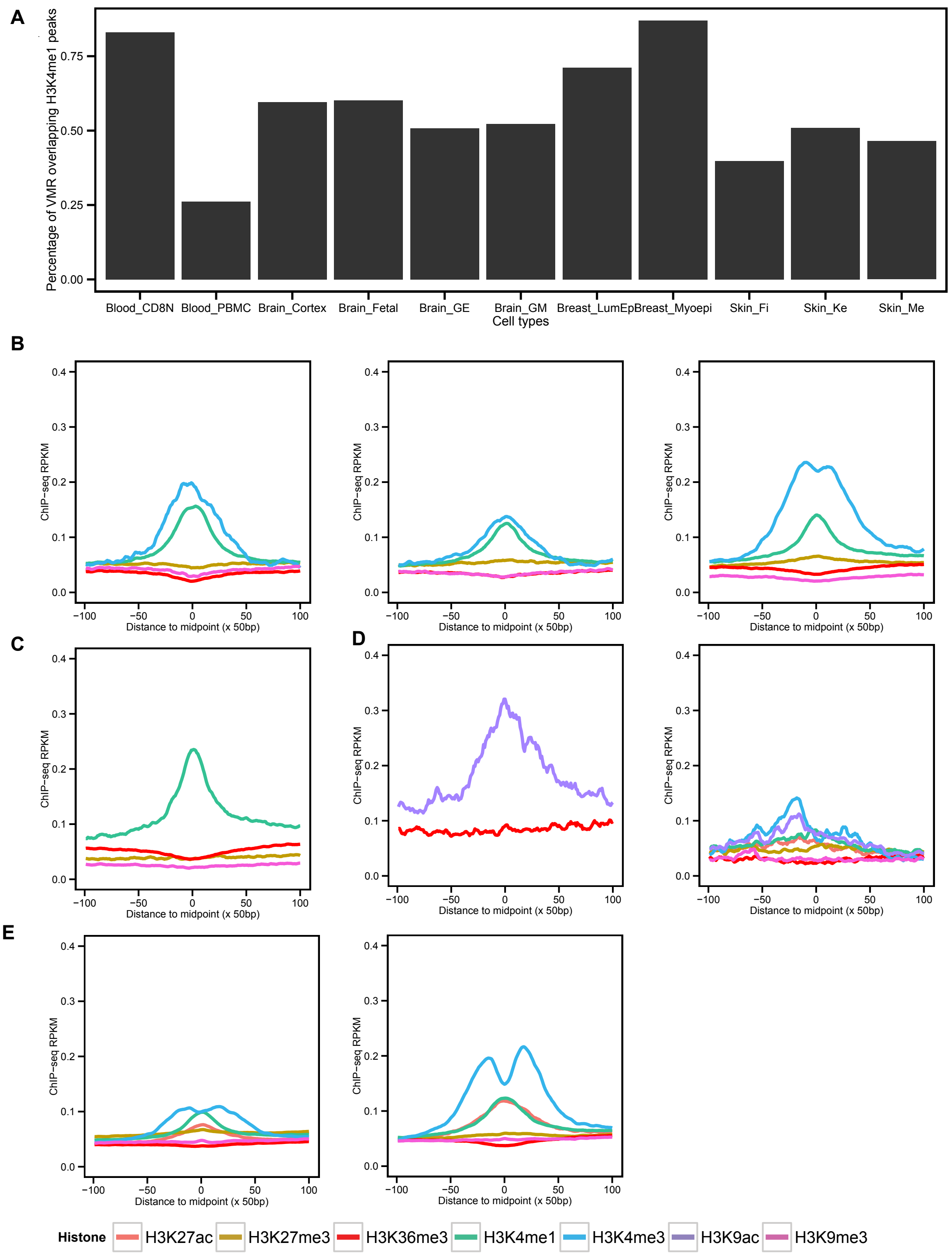
A
Percentage of VMR overlapping H3K4me1 peaks
0.00
0.25
0.50
0.75
Blood_CD8N
Blood_PBMC
Brain_Cortex
Brain_Fetal
Brain_GE
Brain_GM
Breast_LumEp
Breast_Myoepi
Skin_Fi
Skin_Ke
Skin_Me
Cell types
B
C
D
E
ChIP−seq RPKM
Distance to midpoint (x 50bp)
Histone
H3K27ac
H3K27me3
H3K36me3
H3K4me1
H3K4me3
H3K9ac
H3K9me3

Figure S6. Hypomethylated VMRs enrich for enhancer or active transcription histone modifications.

a. Percentage of hypomethylated VMRs overlapping H3K4me1 peaks in different cell types.
b. Mean ChIP-seq signal over 10kb regions centered on the middle point of VMRs in (B) Brain_GE, Brain_GM, Brain_Fetal; (C) Breast_LumEpi; (D) Blood_CD4N, Blood_PBMC; (E) Skin_Fi, Skin_Me.
