# Supplementary material for: Mapping of Variable DNA Methylation Across Multiple Cell Types Defines a Dynamic Regulatory Landscape of the Human Genome
Source: G3 (Bethesda). 2016 Feb 16;6(4):973–86. doi: 10.1534/g3.115.025437 (PMC4825665; doi:10.1534/g3.115.025437)
Supplement: Supplemental Material [file supp_g3.115.025437_FigureS8.pdf]

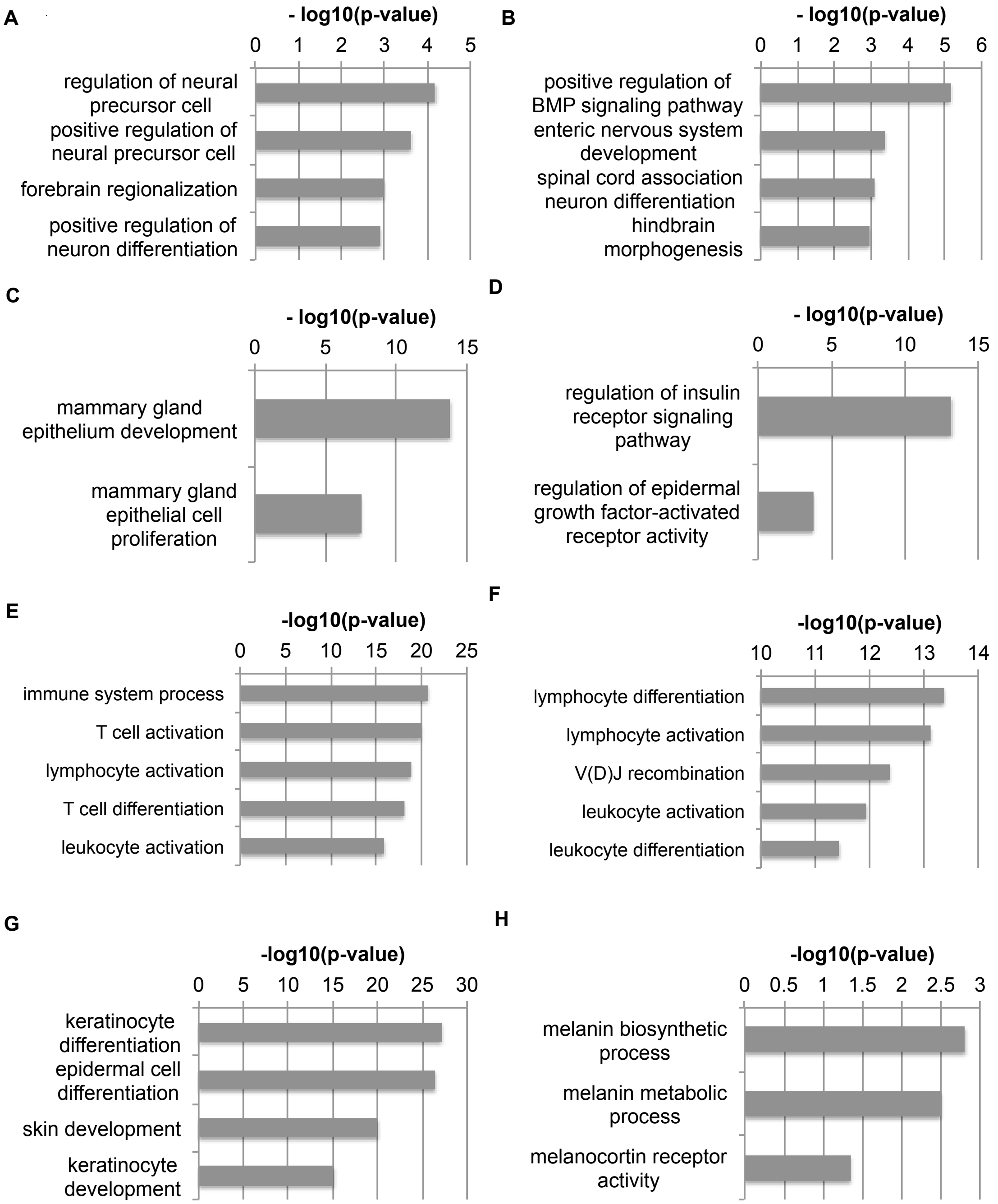

Figure S8. Functional enrichment of genes near hypomethylated VMRs.

Functional enrichment of genes near hypomethylated VMRs in (A) Brain_Cortex (B) Brain_GM (C) Breast_LumEpi (D) Breast_Myoepi (E) Blood_CD8N (F) Blood_CD4N (G) Skin_Ke and (H) Skin_Me.
