# Supplementary material for: Mapping of Variable DNA Methylation Across Multiple Cell Types Defines a Dynamic Regulatory Landscape of the Human Genome
Source: G3 (Bethesda). 2016 Feb 16;6(4):973–86. doi: 10.1534/g3.115.025437 (PMC4825665; doi:10.1534/g3.115.025437)
Supplement: Supplemental Material [file supp_g3.115.025437_FigureS9.pdf]

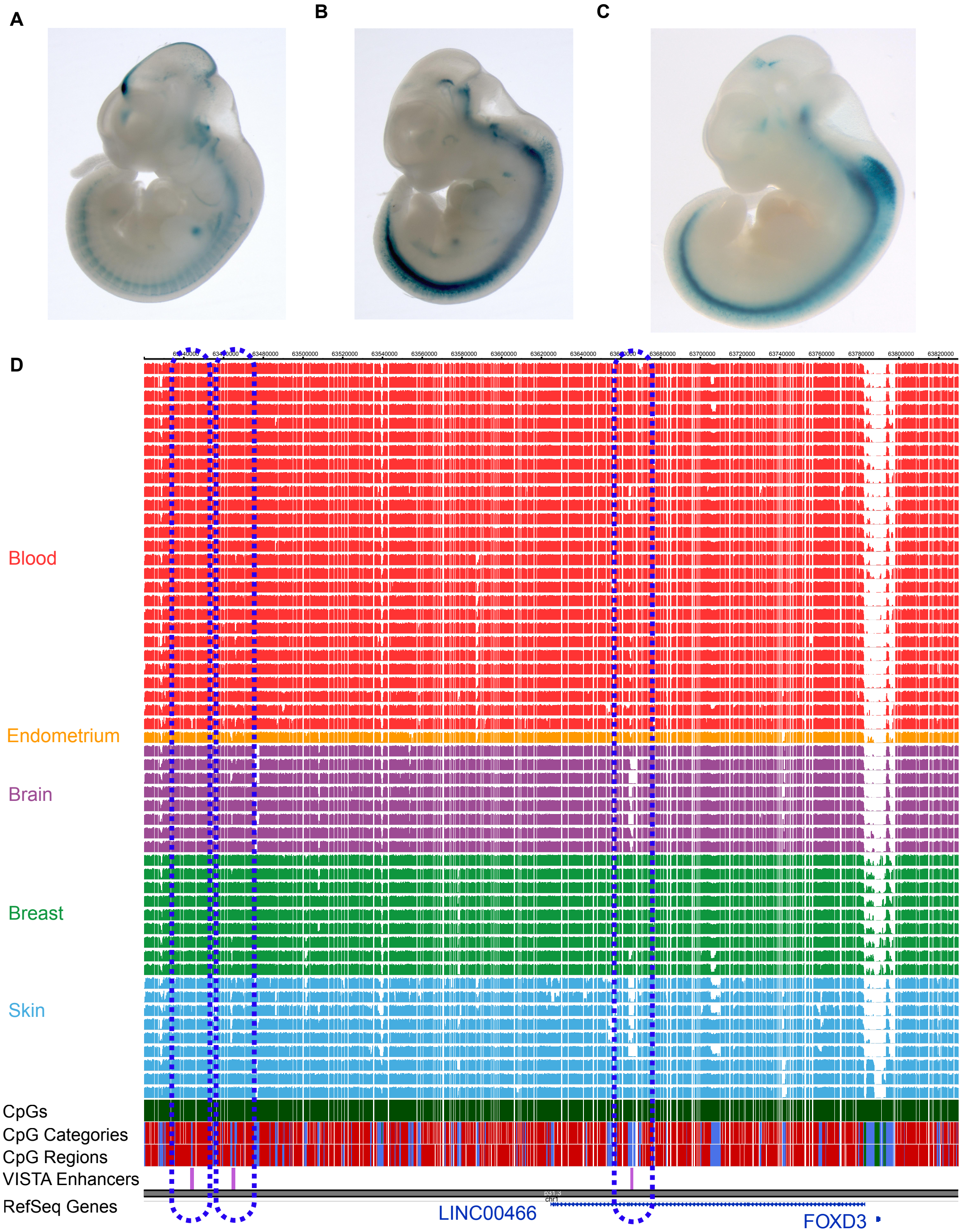

Figure S9. *FOXD3* is potentially regulated by methylation level in nearby regions in brain. Several VMRs upstream of *FOXD3* that show hypomethylation in certain brain cell types overlap with VISTA enhancer elements and VISTA enhancer element (A) hs168 and (B) hs169 and (C) hs764 were validated positive and they lie at 340kb, 320kb and 124kb upstream of *FOXD3* (D).
