# Supplementary material for: Mapping of Variable DNA Methylation Across Multiple Cell Types Defines a Dynamic Regulatory Landscape of the Human Genome
Source: G3 (Bethesda). 2016 Feb 16;6(4):973–86. doi: 10.1534/g3.115.025437 (PMC4825665; doi:10.1534/g3.115.025437)
Supplement: Supplemental Material [file supp_g3.115.025437_FigureS10.pdf]

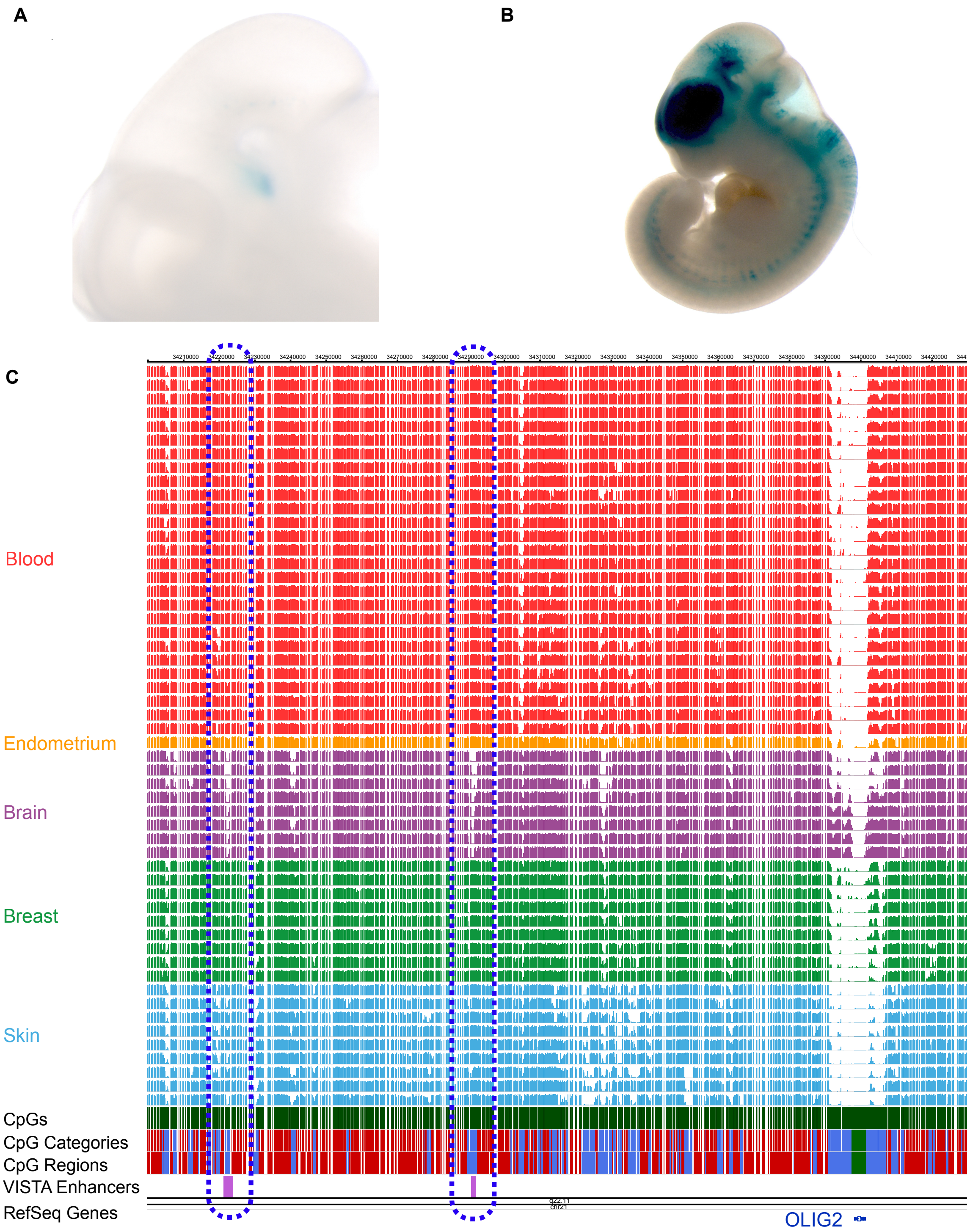

Figure S10. *OLIG2* is potentially regulated by upstream VMRs in fetal brain.

VISTA enhancer element (A) hs1548 and (B) hs1188 were validated as having enhancer function in forebrain in mouse embryo. The browser view of the region upstream of *OLIG2* is shown in (C). Note the hypomethylation in brain.
