# Supplementary figures and images for: Mapping of Variable DNA Methylation Across Multiple Cell Types Defines a Dynamic Regulatory Landscape of the Human Genome
Source: G3 (Bethesda). 2016 Feb 16;6(4):973–86. doi: 10.1534/g3.115.025437 (PMC4825665; doi:10.1534/g3.115.025437)

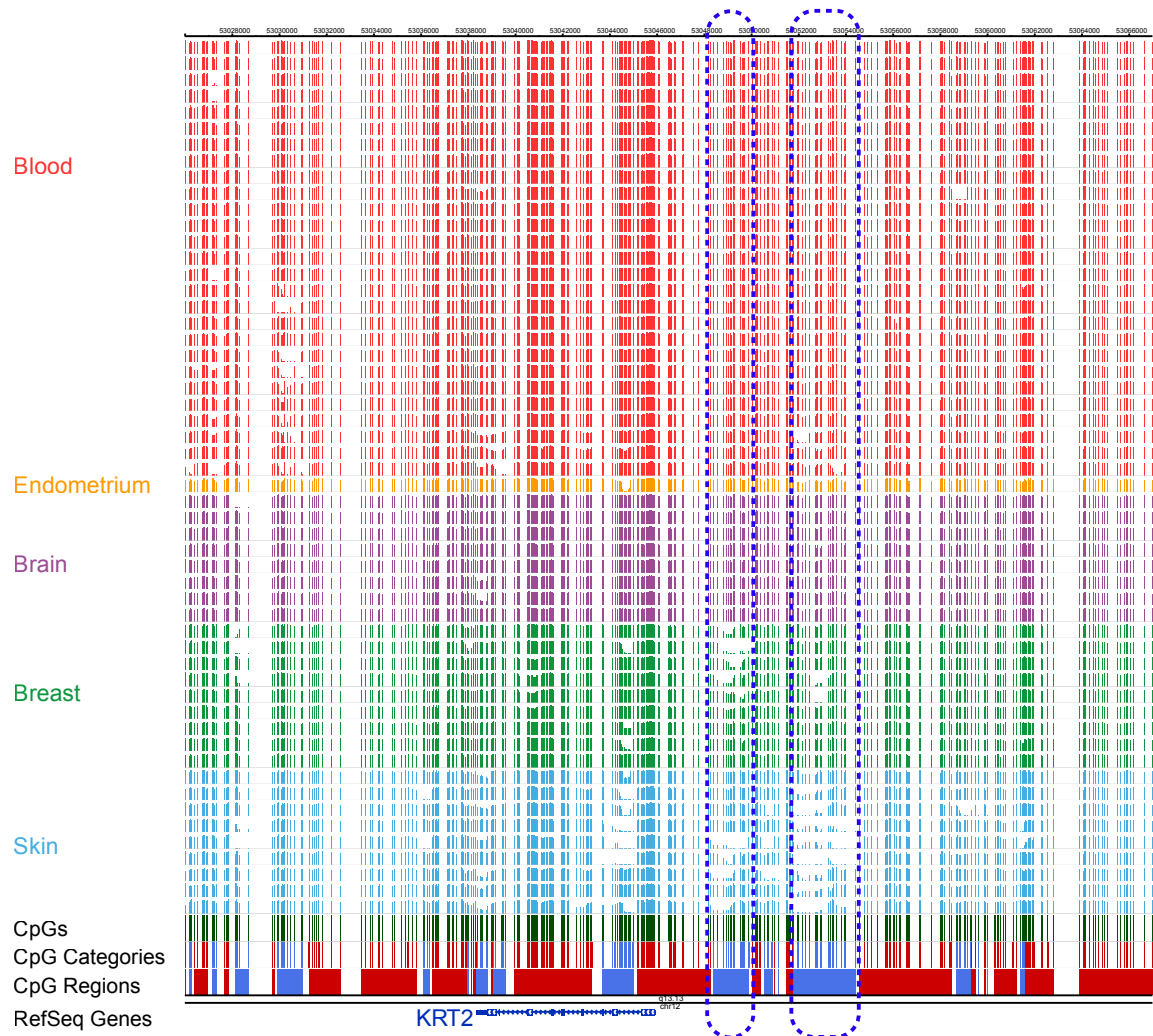

Figure S11. *KRT2* is potentially regulated by upstream VMRs in keratinocyte.

Supplement: Supplemental Material [file supp_g3.115.025437_FigureS11.pdf]

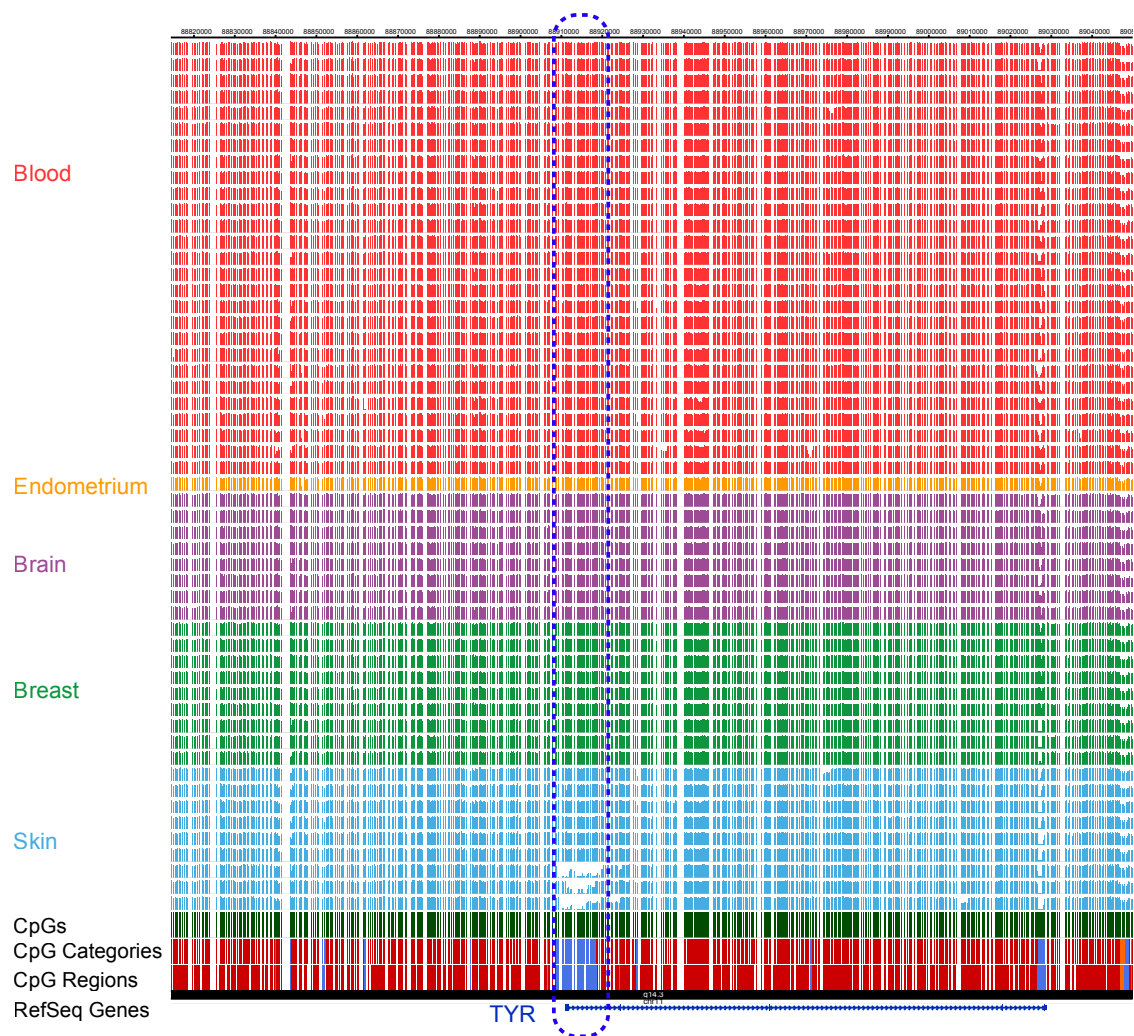

Figure S12. *TYR* is potentially regulated by VMRs in melanocyte.

Supplement: Supplemental Material [file supp_g3.115.025437_FigureS12.pdf]
