# Supplementary material for: Mapping of Variable DNA Methylation Across Multiple Cell Types Defines a Dynamic Regulatory Landscape of the Human Genome
Source: G3 (Bethesda). 2016 Feb 16;6(4):973–86. doi: 10.1534/g3.115.025437 (PMC4825665; doi:10.1534/g3.115.025437)
Supplement: Supplemental Material [file supp_g3.115.025437_FigureS13.pdf]

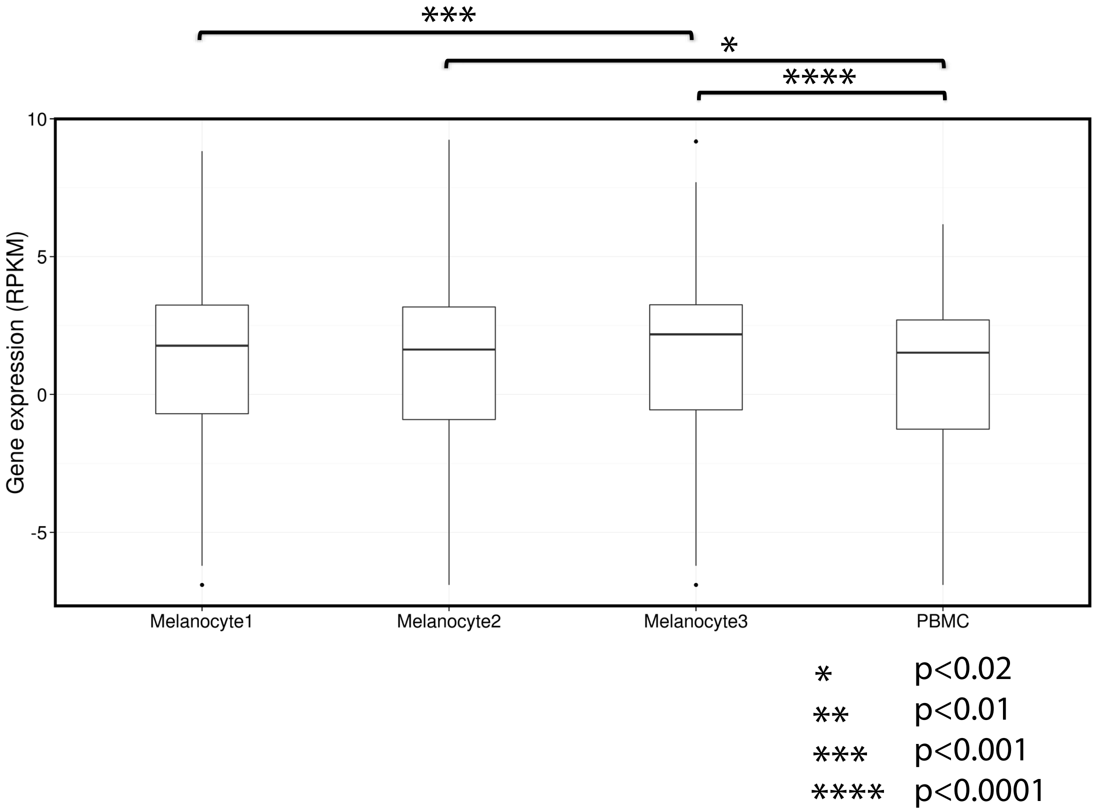

Figure S13. Genes near hypomethylated VMRs show increased expression. RNA-seq derived expression values of genes within 10kb regions of melanocyte hypomethylated VMRs were calculated for melanocyte and PBMC and Kruskal-Wallis test was used to compare expression levels between samples.
