# Supplementary material for: Mapping of Variable DNA Methylation Across Multiple Cell Types Defines a Dynamic Regulatory Landscape of the Human Genome
Source: G3 (Bethesda). 2016 Feb 16;6(4):973–86. doi: 10.1534/g3.115.025437 (PMC4825665; doi:10.1534/g3.115.025437)
Supplement: Supplemental Material [file supp_g3.115.025437_FigureS14.pdf]

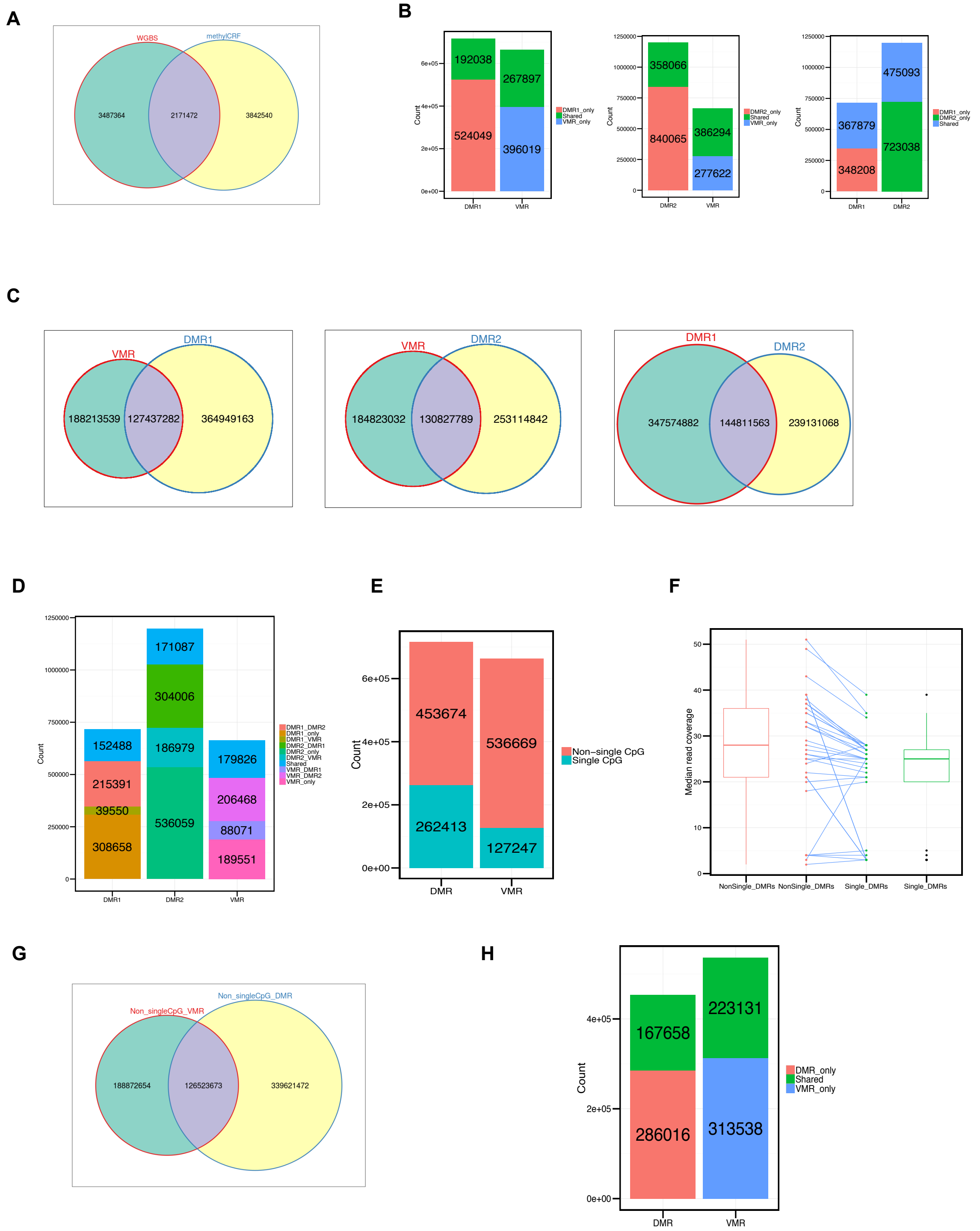
A
WGBS
methylCRF
3487364
2171472
3842540
B
Count
192038
524049
267897
396019
DMR1
VMR
DMR1_only
Shared
VMR_only
358066
840065
386294
277622
DMR2
VMR
DMR2_only
Shared
VMR_only
367879
348208
475093
723038
DMR1
DMR2
DMR1_only
DMR2_only
Shared
C
VMR
DMR1
188213539
127437282
364949163
VMR
DMR2
184823032
130827789
253114842
DMR1
DMR2
347574882
144811563
239131068
D
Count
152488
215391
39550
308658
171087
304006
186979
536059
179826
206468
88071
189551
DMR1
DMR2
VMR
DMR1_DMR2
DMR1_only
DMR1_VMR
DMR2_DMR1
DMR2_only
DMR2_VMR
Shared
VMR_DMR1
VMR_DMR2
VMR_only
E
Count
453674
262413
536669
127247
DMR
VMR
Non−single CpG
Single CpG
F
Median read coverage
NonSingle_DMRs
NonSingle_DMRs
Single_DMRs
Single_DMRs
G
Non_singleCpG_VMR
Non_singleCpG_DMR
188872654
126523673
339621472
H
Count
167658
286016
223131
313538
DMR
VMR
DMR_only
Shared
VMR_only

Figure S14. Comparison between methylCRF predicted VMRs and WGBS predicted DMRs.

A. Overlap between dynamic CpGs identified from WGBS data and variably methylated CpGs identified from methylCRF.
B. Overlap between regions of VMRs and DMRs. VMR refers to the regions defined in this study. DMR1 refers to the regions defined by Ziller et al. and DMR2 by Schultz et al.
C. Overlap between bases covered by VMRs and those by DMRs.
D. Number of regions in each study that overlap with regions defined in none, one or two of the other studies. DMR1_DMR2 indicates overlap between the two studies. DMR1_only indicates regions that are only found in Ziller et al. Shared indicates regions overlap with both other studies.
E. Number of DMRs and VMRs with single CpG and more than one CpG. Only DMRs defined in Ziller et al. was used for analysis in panel E, F, G and H.
F. Median read coverage for each WGBS library on CpGs in single CpG DMRs (Single_DMRs) or non-single CpG DMRs (NonSingle_DMRs).
G. Overlap between bases covered by non-single CpG VMRs and non-single CpG DMRs.
H. Overlap between regions of non-single CpG VMRs and non-single CpG DMRs.
