# Supplementary material for: Mapping of Variable DNA Methylation Across Multiple Cell Types Defines a Dynamic Regulatory Landscape of the Human Genome
Source: G3 (Bethesda). 2016 Feb 16;6(4):973–86. doi: 10.1534/g3.115.025437 (PMC4825665; doi:10.1534/g3.115.025437)
Supplement: Supplemental Material [file supp_g3.115.025437_FigureS15.pdf]

FigS15

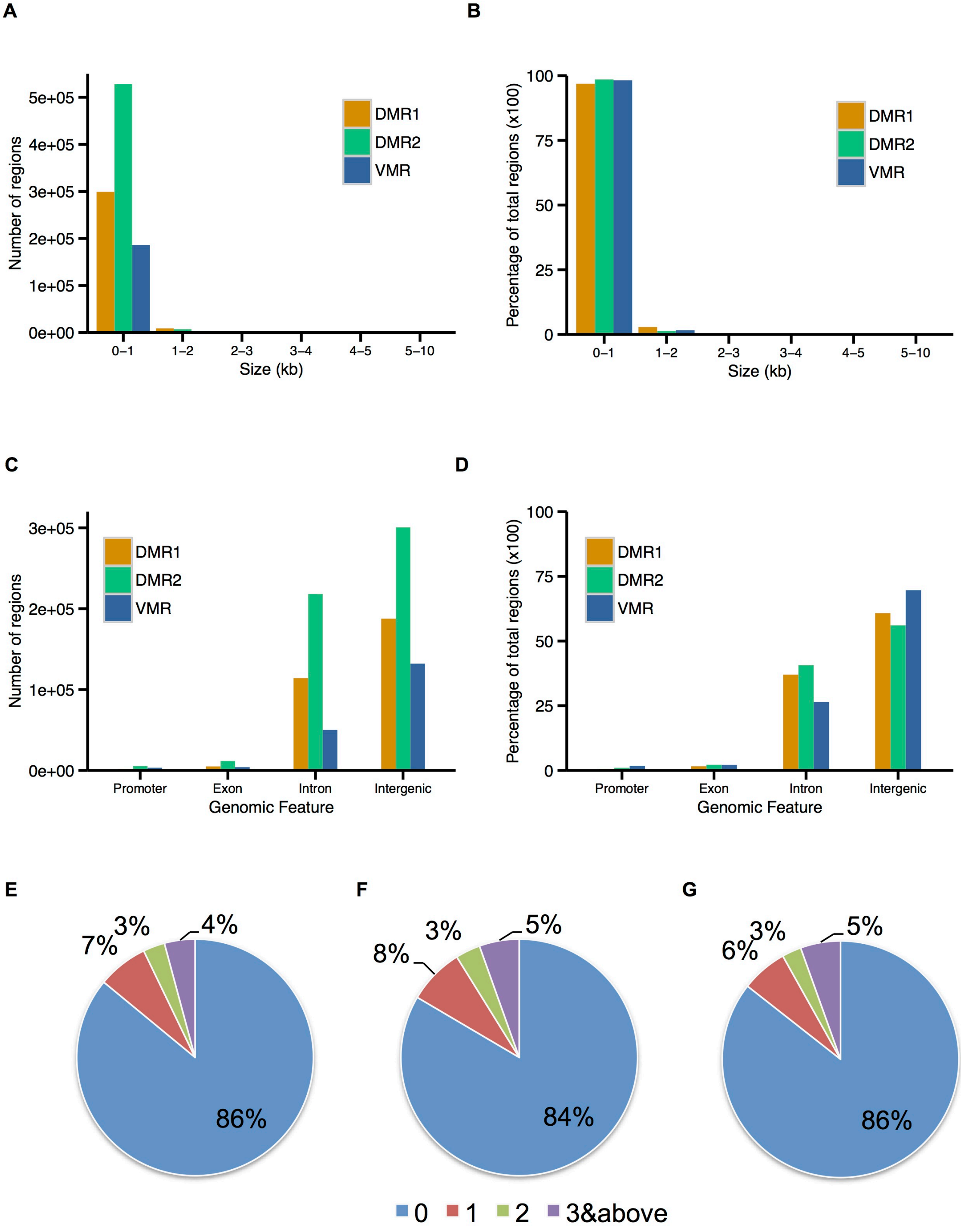
A
B
Number of regions
5e+05
4e+05
3e+05
2e+05
1e+05
0e+00
DMR1
DMR2
VMR
0–1
1–2
2–3
3–4
4–5
5–10
Size (kb)
Percentage of total regions (x100)
100
75
50
25
0
C
D
3e+05
2e+05
1e+05
Promoter
Exon
Intron
Intergenic
Genomic Feature
E
F
G
7%
3%
4%
86%
8%
3%
5%
84%
6%
3%
5%
86%
0
1
2
3&above

Figure S15. Characterization of regions identified specifically in each study.

A. Distribution of region lengths at each range. B. Percentage of regions at each length range. C. Distribution of regions in different genomic features. D. Percentage of regions in genomic features. E-G. Co-localization between regions and transcription factor binding sites. The percentage of regions with 0, 1, 2, or 3 and more transcription factor binding peaks were plotted. E for DMR1 (Ziller et al. study); F for DMR2 (Schultz et al. study); G for VMR (this study).
