# Supplementary material for: Mapping of Variable DNA Methylation Across Multiple Cell Types Defines a Dynamic Regulatory Landscape of the Human Genome
Source: G3 (Bethesda). 2016 Feb 16;6(4):973–86. doi: 10.1534/g3.115.025437 (PMC4825665; doi:10.1534/g3.115.025437)
Supplement: Supplemental Material [file supp_g3.115.025437_FigureS16.pdf]

FigS16

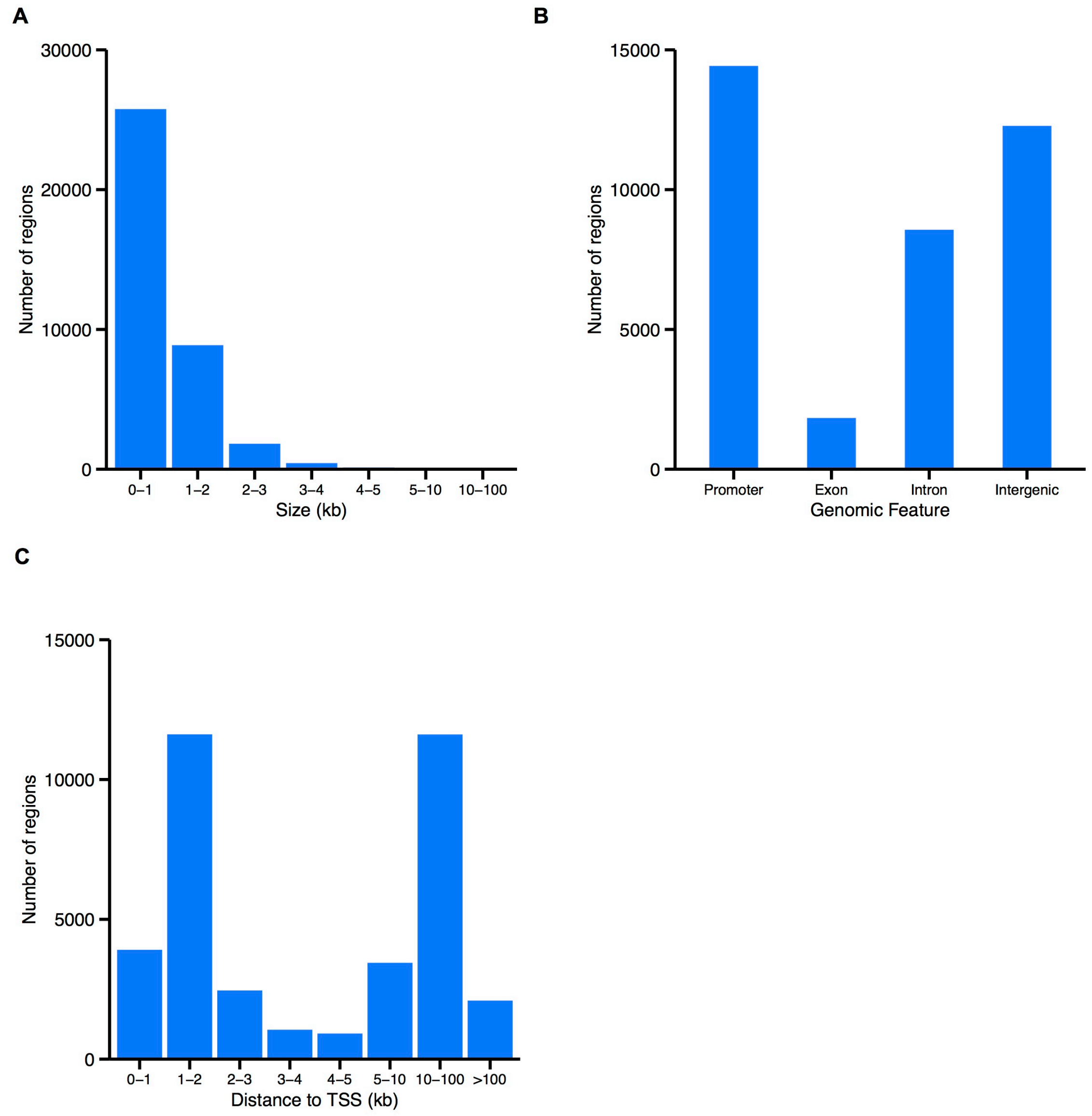

Figure S16. Characterization of constitutively unmethylated regions (UMRs).
A. Distribution of UMR lengths at each range. B. Percentage of UMRs at each length range. C. Distance of UMRs to the nearest transcription start sites (TSSs).
